# PdMoPtCoNi High Entropy Nanoalloy with *d* Electron Self‐Complementation‐Induced Multisite Synergistic Effect for Efficient Nanozyme Catalysis

**DOI:** 10.1002/advs.202406149

**Published:** 2024-08-09

**Authors:** Xuewei Yang, Jianxing Feng, Yuechun Li, Wenxin Zhu, Yifan Pan, Yaru Han, Zhonghong Li, Haijiao Xie, Jianlong Wang, Jianfeng Ping, Wenzhi Tang

**Affiliations:** ^1^ College of Food Science and Engineering Northwest A&F University Yangling Shaanxi 712100 China; ^2^ Department of Chemical Engineering Columbia University New York NY 10027 USA; ^3^ Hangzhou Yanqu Information Technology Co., Ltd Hangzhou Zhejiang 310000 China; ^4^ College of Biosystems Engineering and Food Science Zhejiang University Hangzhou Zhejiang 310058 China

**Keywords:** DFT calculations, heterogeneous catalysis, high‐entropy alloys, Internet of Things, nanozymes

## Abstract

Engineering multimetallic nanocatalysts with the entropy‐mediated strategy to reduce reaction activation energy is regarded as an innovative and effective approach to facilitate efficient heterogeneous catalysis. Accordingly, conformational entropy‐driven high‐entropy alloys (HEAs) are emerging as a promising candidate to settle the catalytic efficiency limitations of nanozymes, attributed to their versatile active site compositions and synergistic effects. As proof of the high‐entropy nanozymes (HEzymes) concept, elaborate PdMoPtCoNi HEA nanowires (NWs) with abundant active sites and tuned electronic structures, exhibiting peroxidase‐mimicking activity comparable to that of natural horseradish peroxidase are reported. Density functional theory calculations demonstrate that the enhanced electron abundance of HEA NWs near the Fermi level (*E*_F_) is facilitated via the self‐complementation effect among the diverse transition metal sites, thereby boosting the electron transfer efficiency at the catalytic interface through the cocktail effect. Subsequently, the HEzymes are integrated with a portable electronic device that utilizes Internet of Things‐driven signal conversion and wireless transmission functions for point‐of‐care diagnosis to validate their applicability in digital biosensing of urinary biomarkers. The proposed HEzymes underscore significant potential in enhancing nanozymes catalysis through tunable electronic structures and synergistic effects, paving the way for reformative advancements in nano‐bio analysis.

## Graphical abstract

As a proof of concept for high‐entropy nanozymes (HEzymes), a class of PdMoPtCoNi nanowires with abundant active sites and tuned electronic structure are designed, and with density functional theory calculations it is demonstrated that their peroxidase‐mimicking activity is comparable to HRP originating from the *d* electrons self‐complementation effect. The HEzymes are combined with a portable electronic device to achieve IoT‐activated POC digital urinalysis.

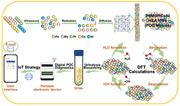

## 1 Introduction

Nanozymes, characterized by their cost‐effectiveness, resilience in harsh catalytic conditions, durability, and recyclability, are regarded as desired alternatives to natural enzymes, representing a transformative strategy to tackle challenges across agriculture, energy, healthcare, and socioeconomics, and serving as a burgeoning area of interest in contemporary biochemistry and physicochemistry research.^[^ ^1^ ^]^ Nevertheless, current constraints on the catalytic performance and tunability of nanozymes arise from their fixed surface atomic site composition and arrangement, posing a critical bottleneck in the advancement of nanozymes technologies.^[^ ^2^ ^]^ Recently, platinum (Pt)‐ and palladium (Pd)‐based nanocrystals have emerged as exemplary noble metal nanozymes due to their exceptional and stable peroxidase‐like (POD‐like) activity in prolonged storage and diverse reaction environments.^[^ ^3^ ^]^ As face‐centered cubic (*fcc*) crystalline structures, Pt and Pd nanozymes primarily mediate the adsorption of H_2_O_2_ substrates, the cleavage of intermediates, and the dissociation of products on major crystal facets such as (1 1 1) or (2 0 0) planes, thereby reducing the energy barrier to mimic the catalytic activity of natural horseradish peroxidase (HRP) in decomposing peroxide substrates.^[^ ^4^ ^]^ As the active sites on the surface of nanozymes are intimately related to their catalytic performance, the catalytic activity of Pt or Pd nanoparticles could be modulated by altering structural features including size, morphology, surface lattice, structural composition, and surface modification.^[^ ^5^ ^]^ The utilization of singular Pt or Pd nanoparticles as catalysts neglects the synergistic effects of different metal crystals on surface configuration and valence electronic structure.^[^ ^6^ ^]^ Thus, inspired by natural enzymes with multimetal active centers, previous studies have extensively investigated the synergistic catalysis strategy of single‐phase alloys to fully exploit the unique adsorption behaviors and electron transfer properties of multimetallic catalysts toward substrates.^[^ ^7^ ^]^ Therefore, a precise alloy design strategy to regulate the geometric configuration and electronic structure of Pt–Pd‐based multimetal systems holds practical significance in enhancing their catalytic performance.

Remarkably, since first reported by Yeh's group in 2004, high‐entropy alloys (HEAs) consisting of ≥5 elements have dramatically expanded the design concept of alloys and received widespread attention as a novel type of catalyst.^[^ ^8^ ^]^ In general, each metal element in HEAs occupies a molar ratio of 5–35%, with the enhanced entropy contribution driving the uniform mixing of multiple atomic species in the crystalline solid‐solution phase.^[^ ^8^, ^9^ ^]^ In contrast to low‐entropy alloys (LEAs), HEAs typically boast better catalytic properties attributed to interatomic *d*‐band ligand effects or crystal lattice strain effects.^[^ ^10^ ^]^ Typically, lattice distortion effects primarily modulate the interfacial adsorption and photoresponsive properties of the materials by optimizing the distribution of transition metal *d* electrons near the Fermi energy level (*E*_F_), which can be reflected by changes in the density of states (DOS) of the crystals.^[^ ^11^ ^]^ Furthermore, accurate control of the structure at the nanoscale to obtain materials with larger specific surface areas and more active sites plays a positive role in advancing the application of HEAs in catalysis.^[^ ^12^ ^]^ Research has demonstrated that Pt atoms can effectively lower the reduction temperature of other metals during the formation of HEA nanomaterials, which enable the simultaneous reduction of different elements at low temperatures, overcoming the difficulties of previous high‐temperature synthesis techniques in fabricating nano‐sized alloys.^[^ ^12^, ^13^ ^]^ Meanwhile, Pd is also commonly used in the design of high entropy alloys due to its remarkable catalytic activity.^[^ ^4^, ^14^ ^]^ Therefore, with the introduction of other *fcc*‐phase transition metals into Pt and Pd, it is theoretically feasible to obtain an ideal HEA (i.e., a stable single‐phase solid solution) with less strain on the crystal structure, and to reduce costs by decreasing the use of noble metals.^[^ ^4^, ^15^ ^]^ To date, HEA nanomaterials are mainly deployed in energy‐related fields (e.g., electrocatalysis), and research in probing their enzyme‐mimicking activity remains at a nascent stage.^[^ ^12^, ^14^, ^16^ ^]^ The self‐complementation effect among the *d* electrons of the constituent transition metals in HEAs would influence the overall distribution of *d* electrons, including the position of the *d*‐band relative to the *E*_F_ and the abundance of *d* electrons near *E*_F_.^[^ ^12^, ^16^ ^]^ These factors would substantially impact the substrate adsorption behavior and electron transfer efficiency of nanozymes during catalysis, which emphasizes the potential enhancement of the catalytic efficiency of nanozymes by cocktail effects.^[^ ^2^, ^17^ ^]^ Additionally, the high thermal stability and corrosion resistance resulting from the high entropy effect could better cope with the harsh conditions that nanozymes may face (e.g., high temperatures, organic solvents, and extreme pH environments), motivating us to explore the feasibility of Pt–Pd‐based HEA nanomaterials as high‐performance nanozymes.^[^ ^1^, ^18^ ^]^

Driven by the concept of HEAs, we first designed and fabricated a class of PdMoPtCoNi HEA nanowires (NWs) as POD mimics with superb catalytic activity and explored the catalytic performance of such mimics using density functional theory (DFT) calculations. The strong binding among the constituent metals facilitates electron transfer at catalytic sites, while the *d* electrons from Co and Ni sites increase the electron density near the *E*_F_, reducing the energy barrier for surface‐to‐substrate transfer during catalysis, thereby enhancing the catalytic performance of HEA NWs. To assess the feasibility of employing HEA NWs as alternatives to HRP, the fabricated high‐entropy nanozymes (HEzymes) are integrated with a custom‐designed portable electronic device that bridges signal conversion and output utilizing the Internet of Things (IoT) strategy for point‐of‐care (POC) digital ultrasensitive diagnostics of urinary biomarkers. Our findings are expected to pioneer new avenues for the rational design of high‐efficiency nanozymes and applications in biosensing.

## 2 Results and Discussion

### 2.1 Preparation and Characterization of the PdMoPtCoNi HEzymes

**Figure 1:**
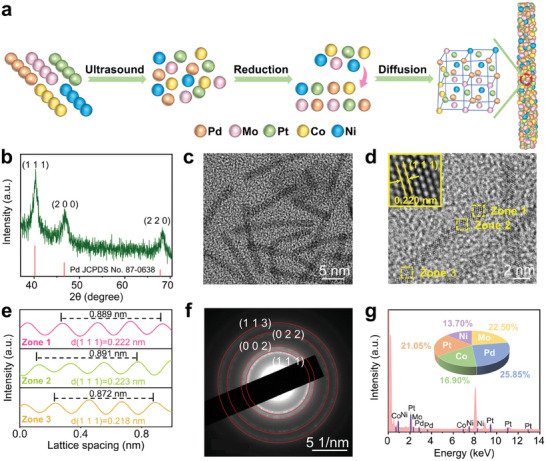
Fabrication and characterization of the PdMoPtCoNi NWs. a) Schematic diagram of the synthesis mechanism of the HEA NWs. b) XRD pattern. c) HRTEM image. d) HRTEM image with a representative atomic arrangement image (inset). e) Integrated pixel intensities of the HEA NWs from Zone 1–3 in d). f) SAED pattern. g) HAADF‐STEM‐EDS characterization. The inset diagram displays the elemental ratios from ICP‐OES.

We synthesized the PdMoPtCoNi HEA nanozymes (HEzymes) via a reduction–diffusion strategy at a low temperature.^[^ ^12b^ ^]^ Typically, five metal precursors including Pt(acac)_2_, Pd(acac)_2_, Mo(CO)_6_, Co(acac)_3_, and Ni(acac)_2_ were concurrently reduced to form single‐phase solid solutions. Concretely, glucose, oleylamine (OAm), and cetyltrimethylammonium bromide (CTAB) were adopted as the reductant, the solvent, and the structure‐direct agent, respectively.^[^ ^16a^ ^]^ The resulting mixture was sonicated and subsequently heated in an oil bath, ultimately yielding single‐phase HEAs driven by the combination of reduction and atomic diffusion (**Figure** **1**a). The obtained X‐ray diffraction (XRD) pattern exhibited three significant diffraction peaks at 40.2°, 46.8°, and 68.3°, assigned to the (1 1 1), (2 0 0), and (2 2 0) facets of Pd (JCPDS No. 87–0638), respectively, indicating that PdMoPtCoNi NWs employed a *fcc* alloy structure instead of multiple phases (Figure 1b).^[^ ^19^ ^]^ The high‐resolution transmission electron microscopy (HRTEM) images in Figure 1c and Figure S1 (Supporting Information) showed that the synthesized HEA NWs possessed an average length of 14.19 nm and an average width of 1.37 nm. The average lattice spacing of the HEA NWs was determined to be 0.220 nm, corresponding to the (1 1 1) crystal planes of Pd to further confirm an *fcc* crystal phase without phase segregation, which was consistent with the XRD result (Figure 1d).^[^ ^20^ ^]^ Notably, the integrated pixel intensities for the (1 1 1) lattices from selected regions exhibited that the average lattice spacing calculated from fast Fourier transform (FFT) analysis varied from 0.218 (Zone 3) to 0.222 (Zone 1), and to 0.223 nm (Zone 2), which demonstrated lattice distortions in the synthesized HEAs (Figure 1e).^[^ ^20^ ^]^ Additionally, TEM images and HRTEM images of the alloys prepared with various reagent additions demonstrated that the increased amount of glucose and Mo(CO)_6_ contributed to obtaining nanowire alloys with smaller sizes and more homogeneous dispersion, suggesting that both glucose and Mo(CO)_6_ acted as reducing agents for morphology and size regulation (Figure 1c; Figures S2 and S3, Supporting Information).^[^ ^12b^ ^]^ The selected area electron diffraction (SAED) of the HEA NWs in Figure 1f also illustrated a typical *fcc* phase, consistent with previous XRD and HRTEM results, which was also in accordance with our previous expectation of selecting elements with moderate nucleation rates to obtain a single‐phase solid solution.^[^ ^12b^ ^]^

The energy dispersive X‐ray spectroscopy (EDS) image and high‐angle annular dark‐field scanning TEM (HAADF‐STEM) with corresponding elemental mapping of the HEAs revealed the distribution of Pd, Mo, Pt, Co, and Ni elements in the NWs, implying the quinary HEAs were successfully prepared (Figure 1g; Figure S4, Supporting Information). The inductively coupled plasma optical emission spectroscopy (ICP‐OES) characterization in Figure 1g also showed that the corresponding atomic ratios of Pd, Mo, Pt, Co, and Ni in the HEA NWs were 26:22:21:17:14, with a conformational entropy of 1.59 R belonging to the high entropy category, and hence the PdMoPtCoNi NWs were classified as high‐entropy nanoscale materials.^[^ ^21^ ^]^ Furthermore, the discrepancy between this atomic ratio and the proportion of precursor salts in the raw material might originate from concentration fluctuations.^[^ ^22^ ^]^ Subsequently, the X‐ray photoelectron spectra (XPS) were adopted as a surface‐sensitive probe to demonstrate the surface states of each element in HEA NWs.^[^ ^14b^ ^]^ Detailed valence data of these five metallic elements are depicted in Figure S5 (Supporting Information). For Pd 3d_5/2_, the peaks ≈335.3 and 336.0 eV are attributed to Pd^0^ and PdO_x, x<1_, respectively. The peaks at 340.5 and 342.0 eV are assigned to the Pd^0^ 3d_3/2_ and PdO_x, x<1_ 3d_3/2_ (Figure S5a, Supporting Information), respectively.^[^ ^14a^ ^]^ In Figure S5b (Supporting Information), the peaks with the binding energies at 231.8 and 234.8 eV can be attributed to Mo^2+^ 3d_5/2_ and Mo^2+^ 3d_3/2_, respectively. The peaks ≈232.1 and 235.5 eV correspond to 3d_5/2_ and 3d_3/2_ of Mo^3+^. The peaks of 71.1 and 71.9 eV are attributed to 4f_7/2_ of Pt^0^ and Pt^2+^, respectively, and the peaks located ≈74.4 and 75.0 eV are attributed to 4f_5/2_ of Pt^0^ and Pt^2+^, respectively (Figure S5c, Supporting Information).^[^ ^19^ ^]^ The Co 2p spectra were split into several peaks, which could be deconvoluted to Co^0^ (2p_3/2_ at 779.2 eV, 2p_1/2_ at 796.1 eV) and Co^2+^ peaks (2p_3/2_ at 781.4 eV, 2p_1/2_ at 798.1 eV) (Figure S5d, Supporting Information).^[^ ^13a^ ^]^ For Ni 2p, Ni^0^ 2p_3/2_ and Ni^2+^ 2p_3/2_ are concentrated at 856.2 and 861.3 eV, respectively. The Ni^0^ 2p_1/2_ and Ni^2+^ 2p_1/2_ peaks are located at 873.9 and 881.8 eV (Figure S5e, Supporting Information). These results indicated that Pd, Pt, Co, and Ni elements in HEA NWs displayed mixed metallic and oxidized valence states.^[^ ^14a^ ^]^ Notably, the Mo element was presented only in the oxidized state, presumably due to that Mo(CO)_6_ not only served as a constituent element in the alloy but also functioned as a reducing agent during the synthesis.^[^ ^12b^ ^]^ Furthermore, optical photographs in Figure S6 (Supporting Information) suggested that the fabricated HEA NWs with a positive zeta potential of 31.6 ± 2.9 mV were stably dispersed in the aqueous solution.

### 2.2 Peroxidase‐Like Catalytic Activity of the HEzymes

**Figure 2:**
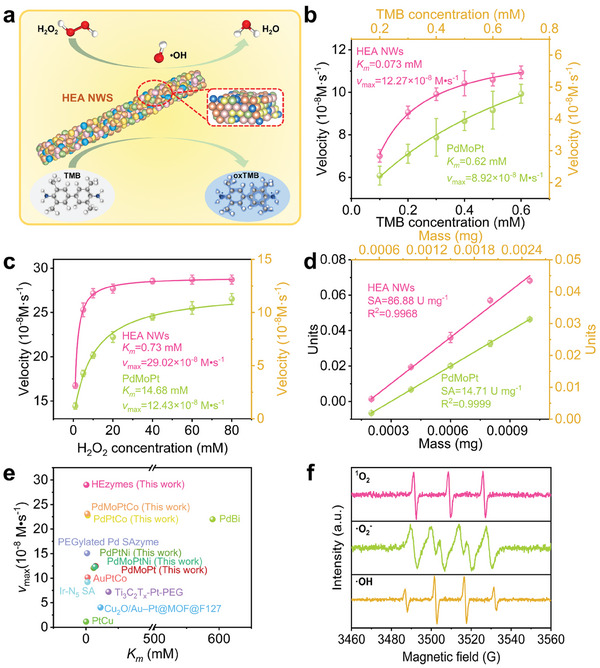
Peroxidase‐like activity of PdMoPtCoNi NWs. a) Schematic illustration of the POD‐like catalytic process of the HEA NWs. Michaelis–Menten curve of the HEA NWs and PdMoPt toward different concentrations of b) TMB and c) H_2_O_2_, respectively. The yellow axes are the parameters corresponding to PdMoPt. d) The specific activity of the HEA NWs and PdMoPt. The yellow axes are the parameters corresponding to PdMoPt. e) Comparison of *K_m_* and *v*_max_ of PdMoPtCoNi HEzymes with other reported catalysts using H_2_O_2_ as substrate. f) ESR spectra of ·OH, ·O_2_^−^, and ^1^O_2_.

To validate the enzymatic performance of the synthesized PdMoPtCoNi NWs, 3,3′,5,5′‐tetramethylbenzidine (TMB) was used as the typical chromogenic substrate.^[^ ^23^ ^]^ As depicted in **Figures** **2**a and S7a (Supporting Information), the blue color and absorption peak at 652 nm originated from the oxidation of TMB due to the decomposition of H_2_O_2_ catalyzed by HEA POD mimics. In addition to TMB, the POD‐like activity of the HEA NWs was further confirmed by observing the oxidation of o‐phenylenediamine (OPD) and 2,2′‐Azino‐bis (3‐ethylbenzothiazoline‐6‐sulfonic acid) diammonium salt (ABTS). Specifically, OPD and ABTS were catalyzed by HEA NWs to generate corresponding yellow and green oxidized products with strong absorption at 431 and 415 nm, respectively (Figure S7b, Supporting Information). To improve the enzyme‐like efficiency of the HEA NWs, catalytic parameters such as pH, temperature, and material concentration were optimized. According to Figure S7c,d (Supporting Information), the POD‐like activity of the HEA NWs was more prominent as compared to the oxidase‐like (OXD‐like) activity with optimal pH and temperature of pH 5.0 and 37 °C, respectively. Furthermore, the oxidation of TMB was highly sensitive to the amount of the HEA NWs in the reaction system, demonstrating the intrinsic POD‐like activity of the HEzymes (Figure S7e, Supporting Information).

To assess the catalytic efficiency of the POD mimics, a steady‐state kinetic assay was conducted by independently varying the concentration of TMB or H_2_O_2_. Typical Michaelis–Menten curves were obtained by plotting the initial reaction rates against the concentration of TMB (Figure 2b) or H_2_O_2_ (Figure 2c). Subsequently, Lineweaver–Burk double reciprocal plots (Figure S8, Supporting Information) demonstrated the Michaelis constants (*K_m_*) and maximum reaction rates (*v*_max_) of the HEA NWs. For comparison, the catalytic performance of PdMoPt, PdPtCo, PdPtNi, PdMoPtCo, and PdMoPtNi POD‐mimics was also investigated. As shown in Figure 2b,c and Figures S9–S14 (Supporting Information), HEA NWs exhibited superior POD‐like activity among the given alloy nanozymes, suggesting that severe lattice distortions endowed HEA NWs with improved catalytic activity compared to conventional alloy nanozymes.^[^ ^8^, ^24^ ^]^ The *K_m_* values (0.073 mm for TMB and 0.73 mm for H_2_O_2_) of HEAs were lower than those of control mimics, HRP, and the majority of nanozymes reported in previous studies (Table S1, Supporting Information), implying superior affinity of the HEAs for both substrates. Additionally, the HEAs also exhibited relatively high *v*_max_ values (12.27 × 10^−8^ m s^−1^ for TMB and 29.02 × 10^−8^ m s^−1^ for H_2_O_2_), indicating the ideal catalytic rate of the HEzymes (Table S1, Supporting Information). Notably, HEA NWs had prominently higher specific activity than control mimics (86.88 U mg^−1^ for HEAs, 14.71 U mg^−1^ for PdMoPt, 30.01 U mg^−1^ for PdPtCo, 19.61 U mg^−1^ for PdPtNi, 33.28 U mg^−1^ for PdMoPtCo and 15.27 U mg^−1^ for PdMoPtNi) and revealed satisfying POD‐like activity after a long period of storage (Figure 2d; Figures S15 and S16, Supporting Information). Overall, the PdMoPtCoNi HEA NWs displayed ideal affinity and catalytic efficiency, which could be identified as a prospective candidate for POD mimics (Figure 2e). To reveal the catalytic mechanism of the POD‐like HEA NWs, ESR spectra were applied to investigate the character of reactive oxygen species (ROS) in the presence of HEA NWs and H_2_O_2_. As demonstrated in Figure 2f and Figure S17 (Supporting Information), the results showed significant ESR signals of radicals including hydroxyl radicals (·OH), superoxide anion (·O_2_^−^), and singlet oxygen (^1^O_2_) with concentrations of 60.9, 42.3, and 35.9 µm respectively, which confirmed the generation of ROS originating from the H_2_O_2_ decomposition catalyzed by the HEzymes.^[^ ^25^ ^]^ Moreover, the decomposition product of H_2_O_2_, ·OH, undergoes self‐disproportionation to yield ^1^O_2_, with a subsequent mutual conversion between ^1^O_2_ and ·O_2_^−^ occurring within the system. This indicates that the formation of ·OH potentially precedes that of ^1^O_2_ and ·O_2_^−^.

### 2.3 DFT Calculations of the HEzymes

**Figure 3:**
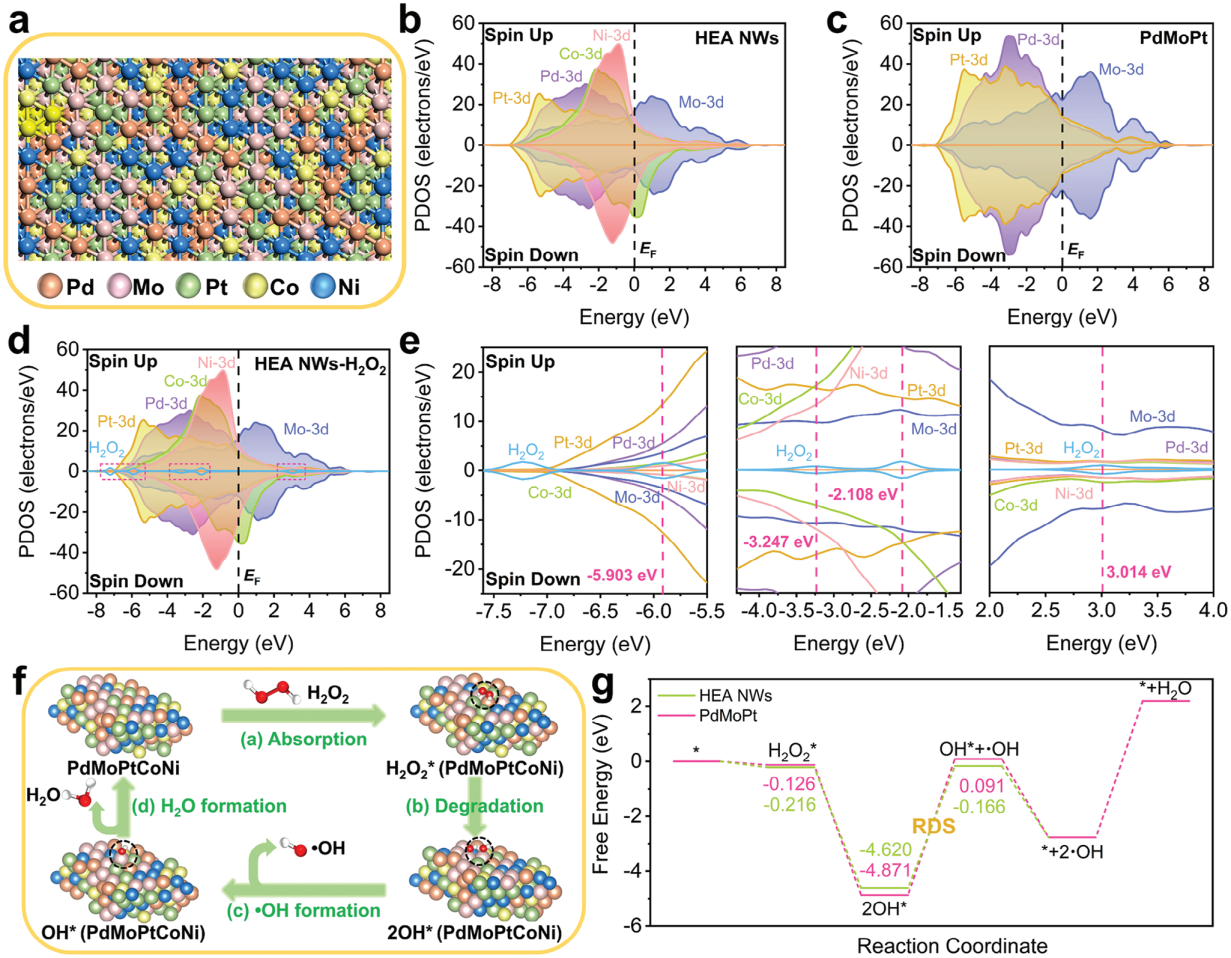
DFT calculations for investigating the proposed catalytic process of the HEA NWs. a) Top view of the optimized geometry of HEA NWs. PODS of b) HEA NWs and c) PdMoPt, respectively. d) PDOS and e) selected enlargements of H_2_O_2_^*^ adsorbed on the HEA NWs surface. f) Proposed reaction process on the HEA NWs. g) Optimized free energy diagrams of the reaction process on the HEA NWs and PdMoPt.

To further elucidate the superior POD‐like activity of HEA NWs, DFT calculations were employed to investigate their electronic structure and catalytic mechanism. According to the characterization results parameters of PdMoPtCoNi, a surface model of the *fcc*‐phase HEAs was established based on the symmetry principle and the random distribution of elements in HEAs (**Figure** **3**a; Figure S18, Supporting Information). Also, the abundant Pd, Mo, and Pt sites on the HEA NWs surface may contribute remarkably to their POD‐like activity. Therefore, an *fcc*‐structured trimetallic PdMoPt alloy nanozyme with a similar structure to that of the HEA NWs was established for comparison (Figures S19 and S20, Supporting Information). Meanwhile, PdPtCo, PdPtNi, PdMoPtCo, and PdMoPtNi alloys were also established to investigate the role of Co and Ni (Figure S21, Supporting Information). Of note, previous studies have shown that the electron‐filling state of the *d*‐band significantly impacts the catalytic process of transition metal sites, especially in the adsorption behavior and electron transfer.^[^ ^14^, ^16^, ^26^ ^]^ The electronic structures of HEA NWs and control alloys were analyzed by calculating the DOS of their *d*‐orbital electrons.^[^ ^27^ ^]^ As shown by the total density of states (TDOS) (Figures S22 and S23, Supporting Information), the HEA NWs and control alloys exhibited a characteristic‐rich distribution of *d* electrons near the *E*_F_, and the partially filled transition‐metal *d* band spanned the *E*_F_. The partial projected density of states (PDOS) (Figure 3b,c; Figure S24, Supporting Information) of HEA NWs and control alloys are calculated to further explore the detailed electronic structure of the *d* orbitals of the constituent elements. Pd, Mo, Pt, Co, and Ni in the HEA NWs displayed distinct *d*‐orbital overlapping near the *E*_F_, indicating the strong bonding among transition metal elements (Figure 3b). Meanwhile, the noticeable *d*‐orbital couplings would enhance the site‐to‐site electron transfer between the different transition metal sites. Among them, the *d*‐band centers of Co and Ni sites were closer to the *E*_F_ and the distribution of *d* electrons was narrower, thus increasing the electron density near the *E*_F_ and leading to the upshift of the overall *d*‐band center of HEAs through the self‐complementation effect, which was more pronounced in the Co sites with a higher degree of spin polarization (Figure 3b).^[^ ^26^, ^28^ ^]^ Compared with control alloys, the contribution of *d* electrons from Co and Ni sites in HEAs significantly increased the electron abundance near the *E*_F_, which could lower the energy barrier for electron transfer from the HEA NWs toward the substrate and improve the catalytic performance of the nanozymes (Figure 3b,c; Figures S9 and S24, Supporting Information). Notably, the higher 3*d* orbital occupancy of Co compared to Ni sites would lead to an upshift of the overall *d*‐band center, which was more favorable for the adsorption of oxygen intermediates. Specifically, PdPtCo and PdMoPtCo exhibited higher electron abundance around *E*_F_ compared to PdPtNi, PdMoPtNi, and PdMoPt, which was particularly noticeable in the spin‐down electron distribution (Figure 3c; Figure S24, Supporting Information). As a result, PdPtCo and PdMoPtCo exhibit higher overall *d*‐band centers relative to *E*_F_ (Figures S22c and S23, Supporting Information), which was more conducive to the adsorption and stabilization of oxygen intermediates. In summary, the incorporation of Co and Ni sites optimized the *d*‐electron distribution of HEA NWs, further contributing to the enzyme‐like activity (Figure S25, Supporting Information). Furthermore, when H_2_O_2_ was adsorbed on the surface of HEA NWs, the *s* orbitals and *p* orbitals of the adsorbed H_2_O_2_ exhibited a distinctive overlap with the 3*d* orbitals of the adjacent transition metal sites, which confirmed the formation of a stable intermediate adsorption state for HEAs and H_2_O_2_ in the reaction (Figure 3d,e).

To further explore the catalytic mechanism of HEA NWs, the explicit and well‐recognized process of ·OH formation was considered the main pathway for the catalytic decomposition of H_2_O_2_ on the surface of POD nanozymes.^[^ ^1^, ^16^, ^29^ ^]^ Figure 3f and Figure S26 (Supporting Information) illustrate the catalytic mechanism of the POD‐like activity of HEA NWs and PdMoPt, respectively. First, an oxygen atom from the free H_2_O_2_ substrate would bond with the HEA NWs at the contact interface to form an H_2_O_2_‐HEAs intermediate complex (H_2_O_2_^*^). Then, the HEA NWs would activate the adsorbed H_2_O_2_, inducing the breaking of the O─O bond through electron transfer resulting in two adsorbed OH (2OH^*^) on the surface of the material. Subsequently, one OH^*^ would be progressively dissociated from the HEAs surface and form the highly reactive, short half‐life ·OH (rate‐determining step, RDS), which would be converted to H_2_O afterward.^[^ ^29^ ^]^ Following the aforementioned reaction mechanism, the POD‐like activity of HEA NWs was specifically explored by calculating the Gibbs free energy changes at each step in the catalytic reaction. As depicted in Figure 3g, HEA NWs and PdMoPt showed similar adsorption energy values for H_2_O_2_ substrates, but HEA NWs displayed higher adsorption energy values for 2OH* (−4.620 eV) and lower free energy at the OH*+·OH step (−0.166 eV). Specifically, according to the Sabatier principle, the substrate‐catalyst intermediate will be unstable if the adsorption strength between the catalyst and substrate or intermediate is too weak. Conversely, if the adsorption strength between them is too strong, it can hinder the dissociation of the intermediate and even lead to catalyst poisoning.^[^ ^30^ ^]^ Compared with PdMoPt, although the adsorption of 2OH^*^ by HEA NWs was relatively weaker, the energy barrier in the dissociation of the first molecule of ·OH was lower (4.454 eV for HEAs, 4.762 eV for PdMoPt). Thus, H_2_O_2_ was thermodynamically more likely to decompose on the HEA NWs surface. Notably, lattice distortions in HEAs would contribute to stabilizing the thermodynamic nonequilibrium states to lower the energy barriers of the reactive species, which also conformed to the relatively lower energy barriers for ·OH dissociation of HEA NWs.^[^ ^31^ ^]^ Thus, the POD‐like activity of HEA NWs was stronger than that of PdMoPt, which was consistent with the results of the enzyme activity measurements. In conclusion, DFT calculations demonstrated the unique electronic structure and catalytic mechanism of HEA NWs and revealed the source of the superb POD‐like activity of HEA NWs, which enhanced further understanding of the structure‐performance relationship of the nanozymes.

### 2.4 Assessment of the Portable Multi‐Channel Colorimetric Electronic Device

**Figure 4:**
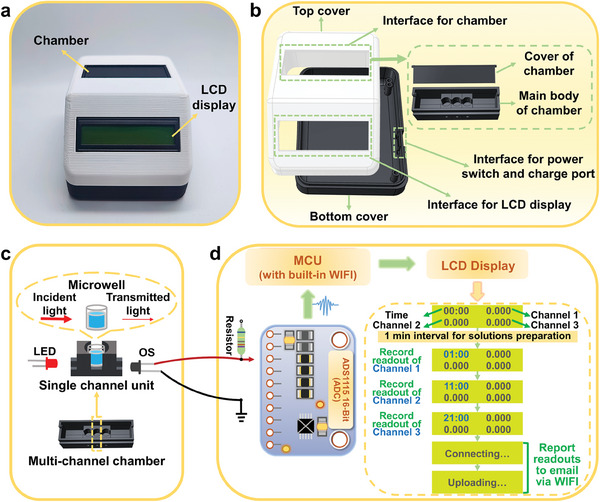
Fabrication of the portable electronic device. a) Photograph of the fabricated device (Length: 11; Width: 9; Height: 7 cm). b) The structure of the device covers and chamber. c) Schematic profile of the optical collection unit. The inset shows the signal light absorption in the chamber. d) Schematic profile of the signal processing unit. The dashed box depicts the process of the device sequentially and automatically recording the concentration of targets and reporting the final result to the user.

Nowadays, digital health driven by portable electronics and IoT strategies provides patients with diagnostic tools and results efficiently.^[^ ^32^ ^]^ Therefore, to enable POC diagnosis of biomarkers, an IoT technology‐facilitated portable colorimetric electronic device was fabricated with three optical channels for multi‐target measurement, which integrated signal conversion, processing, and WIFI transmission (**Figure** **4**a).^[^ ^33^ ^]^ The portable device was composed of three main parts: a 3D printed chamber and reader covers, an optical collection unit for signal conversion, and a readout system for signal processing (Figure 4b–d). As illustrated in Figure 4c, the light‐emitting diodes (LEDs) and the optical sensors (OSs) were responsible for offering the incident light (*I_0_*) and monitoring the transmitted light (*I*) of colored solutions in microwells, respectively. In Figure 4d, the readout system was operated by adopting an analog‐to‐digital converter (ADC) to monitor the response of OSs and utilized an ESP8266 microcontroller unit (MCU) to process the response according to the Lamber–Beer law as shown in Equation (1), and output to the LCD 1602 display. The prepared sample solutions were transferred to an eight‐well ELISA plate strip at 1 min after switching on, where the concentration of the corresponding target was determined for each of the three channels after the successive completion of the chromogenic reaction (Figure 4d).

(1) $$\mathrm{Readout}\;=\;\mathrm{lg}\;\left(I_{0}/I\right)$$

The feasibility of the electronic device employing horizontal incident light for optical inspection was investigated. First, the stability of the readings was assessed for the three channels of colorimetric analysis. As illustrated in Figure S27a (Supporting Information), the results of six repetitive measurements of the three channels remained stable, with relative standard deviations (RSDs) of 0.0045, 0.0077, and 0.0058, respectively, indicating that the instrument possessed satisfactory detection stability. The three channels all adopted LEDs of 652 nm, and therefore the detection variability of different channels was further explored. The results of the F‐test (*p* > 0.05) revealed that no significant difference was found between the detection results of the three channels for both high and low sample concentrations (Figure S27b, Supporting Information). Moreover, the normalized readings of the same solution in the eight microtubes of the plate strip fluctuated between 99.6% and 100.4%, suggesting favorable consistency of the assay performed with different microwells (Figure S27c, Supporting Information). The absorbance measurement performance of the device was further evaluated. According to Figure S27d–f (Supporting Information), the readouts of all three channels for a range of dilutions of the chromogenic solution were linearly proportional to the results of the microplate reader (R^2^ > 0.99), which illustrated the high accuracy of the device for colorimetric measurements. Hence, this portable electronic device exhibited reliable performance in absorbance detection of sample solutions and held great potential for POC monitoring.

### 2.5 Application of the HEzymes‐Based Noninvasive Digital Diagnostics of Urinary Biomarkers

**Figure 5:**
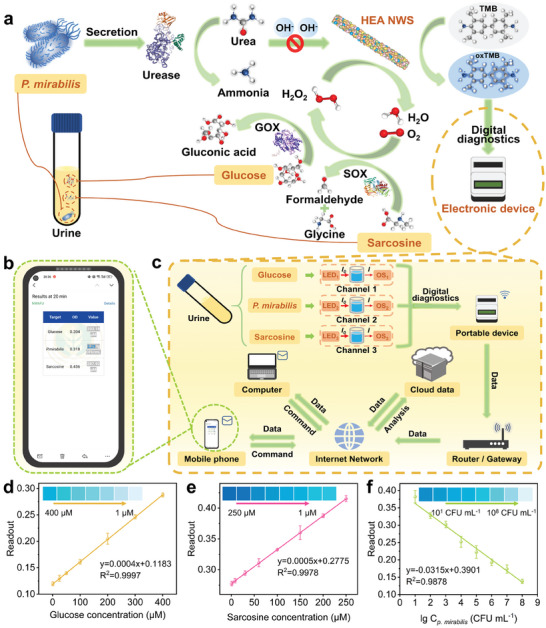
Application of the HEA NWs in POC urinalysis of biomarkers combined with the portable device. a) Versatile applications in digital colorimetric monitoring of glucose, sarcosine, and *P. mirabilis* in urine based on the POD‐like activity of the HEA NWs. (GOX PBD code: 1GAL, [https://www.rcsb.org/structure/1GAL]. Urease PBD code: 2UBP, [https://www.rcsb.org/structure/2UBP]. SOX PBD code: 7EXS [https://www.rcsb.org/structure/7EXS].) b) Enlargement of mobile phone email (user interface) in c). c) Schematic illustration for various biomarkers monitoring via the IoT system. d) Calibration curves of the device readouts vs the concentration of glucose in the TMB/HEA NWs/GOX system. Incubation condition: 15 min at 37 °C. e) Calibration curves of the device readouts vs the concentration of sarcosine in the TMB/HEA NWs/SOX system. Incubation condition: 10 min at 37 °C, then 20 min at room temperature. f) Calibration curves of the device readouts vs the lg concentration of *P. mirabilis* in the H_2_O_2_/TMB/HEA NWs/urea system. Incubation condition: 10 min at 37 °C, then 10 min at room temperature. All data are presented as mean ± SD (*n* = 3 independent samples).

With the advancement of POC diagnostics, digital monitoring of biomarkers gradually reveals potential for the accurate prediction and diagnosis of a variety of diseases.^[^ ^34^ ^]^ Particularly, urine is a class of easily sampled and noninvasive biofluids containing various substances such as metabolites and microbial analytes that have been proven to be valuable in the prediction, clinical diagnosis, and prognosis of kidney diseases, leading to the attraction of monitoring urinary biomarkers.^[^ ^32^, ^35^ ^]^ Diabetes mellitus is a common noncommunicable disease characterized by chronic elevation of blood glucose levels, which can cause dramatic fluctuations that pose a serious threat to public health. The detection of urinary glucose allows an easier and safer indication of the risk of developing diabetes.^[^ ^36^ ^]^ As a clinical marker of early prostate cancer (PCa), the level of sarcosine rises dramatically as the disease worsens, thus the monitoring of urinary sarcosine levels plays an essential function in the identification of PCa.^[^ ^37^ ^]^ *Proteus mirabilis* (*P. mirabilis*) can secret urease to hydrolyze urea, which may ultimately lead to urinary tract infections due to the further formation of bladder or kidney stones derived from urine alkalinization.^[^ ^38^ ^]^ To verify the potential application of the HEzymes featured remarkable catalytic performance combined with the portable device to achieve urine POC bio‐diagnose, colorimetric detection of glucose, sarcosine, and *P. mirabilis* was performed (**Figure** **5**a). Notably, the cost‐effective device was equipped with a wireless data transmission function to automatically report test results to an E‐mail address based on IoT strategy as depicted in Figure 5c.^[^ ^39^ ^]^ Specifically, diagnosis results from the device were automatically transmitted and collected in the form of cloud data via a global network for further emailing to the user (Figure 5b,c).

For glucose detection, glucose is catalyzed to form H_2_O_2_ with the aid of glucose oxidase (GOX), which can produce ROS to function as the substrate of TMB oxidation (Figure 5a). Thus, the readout elevated as more blue color oxTMB was generated with increasing glucose concentration (Figure 5d). As depicted in Figure 5d and Figure S28a (Supporting Information), the absorbance at 652 nm exhibited a superior linear relationship (R^2^ = 0.9997) with glucose concentration ranging from 1–400 µm, and the limit of detection (LOD) was determined to be 0.41 µm based on the 3*N*/*S* principle (*N*: the standard deviation of the blank group; *S*: the slope of the standard curve). Similar to glucose, sarcosine can produce H_2_O_2_, glycine, and formaldehyde after recognition by the sarcosine oxidase (SOX), and the generated H_2_O_2_ is involved in the oxidation of TMB with catalysis by HEzymes (Figure 5a). Likewise, the blueness of the reaction system deepened with the increased concentration of sarcosine. The detection of sarcosine featured the dynamic range and the LOD (3*N*/*S*) of 1–250 and 0.32 µm with a satisfactory correlation coefficient (R^2^ = 0.9978), respectively (Figure 5e; Figure S28b, Supporting Information). For *P. mirabilis* monitoring, a rapid urease‐based assay in the urine is reliable for the prevention of stone formation since urease cannot be produced by the human body.^[^ ^40^ ^]^ Specifically, the NH_3_ generated from the decomposition of urea by urease up‐regulates the pH of the reaction system thereby depressing the mimetic enzyme activity of HEzymes, and thus a pH‐mediated urease colorimetric sensing platform could be constructed (Figure 5a). According to Figure S28c,d (Supporting Information), the absorbance at 652 nm decreased linearly (R^2^ = 0.9881) with the increase of urease concentration over the range of 3–150 mU mL^−1^, and the LOD (3*N*/*S*) was calculated to be 1.13 mU mL^−1^. Based on the above concept, the proposed assay platform could be applied in *P. mirabilis* detection. As the concentration of *P. mirabilis* increased from 10^1^ to 10^8^ CFU mL^−1^, the POD‐like activity of HEzymes gradually decreased resulting in lower production of oxTMB, and the regression equation was described as Readout = −0.0315 lg (*P. mirabilis*) + 0.3901 (R^2^ = 0.9878) with the LOD of 1.81 CFU mL^−1^ (3*N*/*S*) (Figure 5f; Figure S28e, Supporting Information). Benefiting from the remarkable enzymatic activity, the HEzymes showed significant advantages in detection performance, which enabled ultrasensitive urinalysis monitoring with lower LODs compared to other catalysts (Table S2, Supporting Information).

The interference of potential substances on the chromogenic system was investigated, such as urine‐relevant ions (Na^+^, K^+^, and NH_4_^+^), amino acids (Alanine, Arginine, Leucine, and Tryptophan), and sugars (Fructose, Lactose, Glucose, and Sucrose). As shown in Figure S29a (Supporting Information), the effect of interfering substances on the HEzymes/TMB/H_2_O_2_ system was negligible except NH_4_^+^, suggesting promising selectivity for the analysis. Furthermore, the selectivity of *P. mirabilis* detection was verified by testing a series of other bacteria including *Staphylococcus aureus* (*S. aureus*), *Escherichia coli* (*E. coli)*, and *Listeria monocytogenes* (*L. monocytogenes*). In comparison to the other three pathogens, only *P. mirabilis* induced a remarkable decrease in absorbance, which indicated the favorable selectivity of the colorimetric platform for *P. mirabilis* (Figure S29b, Supporting Information).^[^ ^40^ ^]^ To demonstrate the potential application in clinical diagnosis, we further investigated the assay in artificial urine samples. The artificial urine was diluted 50‐fold to effectively reduce matrix interference for glucose, sarcosine, and *P. mirabilis* analysis.^[^ ^41^ ^]^ As illustrated in Table S3 (Supporting Information), the recovery of glucose detection ranged from 101.59% to 117.34% with a relative standard deviation (RSD) of 3.08%–9.09%, further indicating the applicability of urine sample monitoring. For sarcosine monitoring, the favorable recovery rate (85.69%–103.67%) and RSD (7.10%–9.71%) suggested practical application in real sample testing (Table S3, Supporting Information). Also, the recovery rate and RSD obtained for *P. mirabilis* detection in urine samples were 82.78%−113.29% and 7.31%−8.64% respectively (Table S3, Supporting Information), which demonstrated the ideal practicality of this assay. Furthermore, the stability and repeatability of these assays were confirmed to be excellent based on the corresponding tests (Figure S30a–f, Supporting Information). Remarkably, the testing was assumed to be conducted at or near patient sittings with the aid of the portable electronic device incorporated with automatic reporting of results. Specifically, the device automatically recorded the readouts of channels 1 (Glucose), 2 (*P. mirabilis*), and 3 (Sarcosine) after 0, 10, and 20 min of color development at room temperature respectively, and reported the recorded readouts and the converted concentrations to the user interface by E‐mail (Figure 4d).^[^ ^42^ ^]^ The easy‐to‐use operation of the device and the detailed content of the E‐mail are shown in Video S1 (Supporting Information) and Figure S31 (Supporting Information), respectively. Overall, colorimetric sensing strategies based on HEzymes and portable electronics provided an effective tool for the digital monitoring of urinary biomarkers, highlighting the potential of digital health.

## 3 Conclusion

In summary, we have successfully fabricated a class of novel PdMoPtCoNi HEzymes with remarkable substrate affinity and catalytic velocity. The distinctive composition of transition metal sites and the moderate lattice distortion in HEA NWs influence the surface chemical state, thereby promoting the adsorption, electron transfer, and dissociation of the H_2_O_2_ substrate at the material interface. DFT calculations elucidated the catalytic mechanism of HEA NWs and contributed to the understanding of the essential role of electronic structure in enzyme‐like activity. The strong *d*‐orbital coupling of the constituent metals in the HEAs alters the overall electron distribution near the *E*_F_, which facilitates electron transfer efficiency during catalysis. Also, the *d* electrons from Co and Ni sites markedly increase the electron abundance near the *E*_F_, optimizing the energy barrier for OH* dissociation on PdMoPt and thus improving the catalytic performance. As a demonstration, the synthesized HEzymes were employed as biosensors in conjunction with a 3D‐printed portable electronic device developed based on IoT technology, enabling digital POC screening of glucose, sarcosine, and *P. mirabilis* in urine. In conclusion, the POD mimics proposed in this study aimed to broaden the design of high‐efficiency nanozymes and provided new perspectives for the nano‐biosensing application of HEAs in digital health.

## Conflict of Interest

The authors declare no conflict of interest.

## Supporting information

Supporting Information

Supplemental Video 1

## Data Availability

The data that support the findings of this study are available from the corresponding author upon reasonable request.
