# Supplementary material for: PdMoPtCoNi High Entropy Nanoalloy with d Electron Self‐Complementation‐Induced Multisite Synergistic Effect for Efficient Nanozyme Catalysis
Source: Adv Sci (Weinh). 2024 Aug 9;11(38):2406149. doi: 10.1002/advs.202406149 (PMC11481210; doi:10.1002/advs.202406149)
Supplement: Supplementary file 1 — Supporting Information [file ADVS-11-2406149-s002.docx]

**Supporting Information**

**PdMoPtCoNi High Entropy Nanoalloy with *d* Electron Self-Complementation-Induced Multisite Synergistic Effect for Efficient Nanozyme Catalysis**

*Xuewei Yang^1^, Jianxing Feng^1^, Yuechun Li^1^, Wenxin Zhu^1^, Yifan Pan^1^, Yaru Han^2^, Zhonghong Li^1^, Haijiao Xie^3^,* *Jianlong Wang*^1^, Jianfeng Ping*^4^, Wenzhi Tang*^1^*

^1^College of Food Science and Engineering, Northwest A&F University, 22 Xinong Road, Yangling 712100, Shaanxi, China. ^2^Department of Chemical Engineering, Columbia University, New York, NY 10027, USA. ^3^Hangzhou Yanqu Information Technology Co., Ltd. Hangzhou 310000, Zhejiang, China. ^4^College of Biosystems Engineering and Food Science, Zhejiang University, Hangzhou 310058, Zhejiang, China.

*Corresponding author.

E-mail: tangwenzhi@nwsuaf.edu.cn, jfping@zju.edu.cn, [wanglong79@nwsuaf.edu.cn](mailto:wanglong79@nwsuaf.edu.cn)

**This PDF file includes:**

Experimental Section

Supplementary Tables S1-S3

Supplementary Figures S1-S31

**Table of Contents**

**Experimental Section** S5

**Table S1.** Comparison of the kinetic parameters of PdMoPtCoNi HEzymes with PdMoPt, PdPtCo, PdPtNi, PdMoPtCo, PdMoPtNi, HRP, and previously reported nanozymes S10

**Table S2.** Comparison of the analytical performance of the proposed colorimetric strategy with previously reported assays S11

**Table S3.** Detection of glucose, *P. mirabilis*, and sarcosine in urine samples S12

**Figure S1.** Size distribution of the HEA NWs S13

**Figure S2.** TEM images of the products synthesized with Mo(CO)_6_ (20 mg), glucose (10 mg), and CTAB (128 mg) S14

**Figure S3.** TEM images of the products synthesized with Mo(CO)_6_ (20 mg), glucose (60 mg), and CTAB (128 mg) S15

**Figure S4.** EDS Mapping characterization of the HEA NWs S16

**Figure S5.** XPS spectra of (a) Pd 3d, (b) Mo 3d, (c) Pt 4f, (d) Co 2p, and (e) Ni 2p for the HEA NWs S17

**Figure S6.** Images of the HEA NWs aqueous solutions at room temperature with various storage days S18

**Figure S7.** The POD-like activity of the HEA NWs S19

**Figure S8.** Lineweaver-Burk plots of the HEA NWs with (a) TMB and (b) H_2_O_2_ in HAc-NaAc buffer (0.1 M, pH 5.0) at room temperature. S20

**Figure S9.** Comparison of the POD-like activity S21

**Figure S10.** Lineweaver-Burk plots of the PdMoPt with (a) TMB and (b) H_2_O_2_ in HAc-NaAc buffer (0.1 M, pH 5.0) at room temperature. S22

**Figure S11.** Enzyme kinetic performance of the PdPtCo S23

**Figure S12.** Enzyme kinetic performance of the PdPtNi S24

**Figure S13.** Enzyme kinetic performance of the PdMoPtCo S25

**Figure S14.** Enzyme kinetic performance of the PdMoPtNi S26

**Figure S15.** Specific activity of the PdPtCo, PdPtNi, PdMoPtCo, and PdMoPtNi S27

**Figure S16.** Investigation of the POD-like activity of HEA NWs at room temperature in different storage times. S28

**Figure S17.** Concentration histogram of·OH, ·O_2_^-^ and ^1^O_2_ S29

**Figure S18.** Construction of the HEA NWs model S30

**Figure S19.** Top view of the optimized geometry of PdMoPt S31

**Figure S20.** Construction of PdMoPt model S32

**Figure S21.** Construction of PdPtCo, PdPtNi, PdMoPtCo, and PdMoPtNi model S33

**Figure S22.** TDOS of (a) HEA NWs, (b) HEA NWs with H_2_O_2_ adsorbed on the surface, and (c) PdMoPt S34

**Figure S23.** TDOS of (a) PdPtCo, (b) PdPtNi, (c)PdMoPtCo, and (d)PdMoPtNi S35

**Figure S24.** PDOS of (a) PdPtCo, (b) PdPtNi, (c)PdMoPtCo, and (d)PdMoPtNi S36

**Figure S25.** Comparison of *d*-band center and enzymatic activity S37

**Figure S26.** Proposed reaction process on PdMoPt S38

**Figure S27.** Evaluation of the portable electronic device S39

**Figure S28.** Detection of urinary biomarkers S40

**Figure S29.** Selectivity analysis S41

**Figure S30.** Assessment of stability and repeatability of detection S42

**Figure S31.** Detailed content of the E-mail S43

**Experimental Section**

**Materials.** Oleylamine (OAm) and molybdenum hexacarbonyl (Mo(CO)_6_, 98%) were purchased from Macklin Biochemical Technology Co., Ltd (Shanghai, China). 3,3′,5,5′-tetramethylbenzidine (TMB), o-phenylenediamine (OPD), sarcosine, urea, and other metal precursors were supplied by Shanghai Aladdin Biochemical Technology (Shanghai, China), including cobalt (III) acetylacetonate (Co(acac)_3_, 98%), nickel (II) acetylacetonate (Ni(acac)_2_, 97%), platinum (II) acetylacetone (Pt(acac)_2_, 97%), palladium (II) acetylacetonate (Pd(acac)_2_, 99%). 2,2′-Azino-bis (3-ethylbenzothiazoline-6-sulfonic acid) diammonium salt (ABTS) and cetyltrimethylammonium bromide (CTAB) were obtained from Shanghai Sigma-Aldrich Trading Co., Ltd (Shanghai, China). Glucose and hydrogen peroxide (H_2_O_2_, 30%) were purchased from Guangdong Guanghua Technology Co., Ltd (Guangdong, China). Urease was acquired from Beijing Solarbio Science & Technology Co., Ltd (Beijing, China). Glucose oxidase and sarcosine oxidase were purchased from Shanghai Yuanye Bio-Technology Co., Ltd. (Shanghai, China). Tryptone soy broth (TSB), Luria-Bertani (LB) nutrient agar, and LB broth were obtained from Beijing Land Bridge Technology Co., Ltd (Beijing, China).

**Preparation of PdMoPtCoNi HEA NWs.** PdMoPtCoNi HEA NWs were prepared based on the previous literature with a little modification.^[12b]^ Typically, 128 mg of CTAB was dissolved in 8 mL OAm in a flask under 30 min of ultrasonication. Subsequently, Pt(acac)_2_ (10 mg), Pd(acac)_2_ (5.1 mg), Mo(CO)_6_ (50 mg), Co(acac)_3_ (6 mg), Ni(acac)_2_ (4 mg) were added and sonicated with glucose for 2 h to acquire a homogeneous mixture. The mixture was allowed to heat at 80 ºC for 5 min under magnetic stirring in an oil bath and then heated to 220 ºC and kept at this temperature for 2 h. The final black products were collected by centrifugation and washed four times with an ethanol/cyclohexane (v:v = 1:1) mixture. After that, the precipitates were redispersed in ethanol for further experiments.

**Characterization of PdMoPtCoNi HEA NWs.** TEM imaging, HRTEM imaging, and SAED analysis were obtained by a JEM 2100 instrument (JEOL, Japan) operated at an accelerating voltage of 200 kV. EDS elemental mapping was conducted on an X-max 100TLE (Oxford, UK). Powder XRD was performed by a D8 Advance X-ray diffractometer (Bruker, Germany). XPS spectra were collected on an Axis Ultra DLD spectrometer (Kratos Analytical, UK) with a monochromatic Al K𝛼 X-ray source (1486.6 eV). The elemental compositions of HEA NWs were acquired by ICP-OES (720ES, Agilent, US). Zeta potential was measured by ZEN3600 Zetasizer Nano (Malvern, England). UV-vis spectra were obtained by using a UV-2550 UV-vis spectrophotometer (Shimadzu, Japan). Absorbance at 652 nm was recorded by a Multiskan MK3 microplate reader (Thermo Fisher Scientific, US) and fabricated device.

**Catalytic Performance of PdMoPtCoNi HEA NWs.** The POD-mimic activity of HEA NWs was determined by evaluating the catalytic effect on TMB in the presence of H_2_O_2_.^[23]^ Briefly, H_2_O_2_ (10 mM), TMB (1.0 mM), and HEA NWs (5 μg mL^-1^) were added into HAc-NaAc buffer (0.1 M, pH 5.0) and incubated at room temperature for 5 min, followed by measuring the absorbance of the systems at 652 nm. Also, the OXD-mimic activity of HEA NWs was accessed in the absence of H_2_O_2_ addition. The conditions of the enzyme-like activity of HEA NWs were optimized by setting a series of pH values (2.0 to 7.0) and temperature (25 to 70 ºC) of the reaction. To obtain the optimal incubation time and HEA NWs concentration for the reaction, the time-dependent kinetics were also investigated based on accessing the TMB-derived oxidation product.

**Enzyme Kinetic Testing of** **PdMoPtCoNi HEA NWs.** Steady-state kinetic assays were conducted in HAc-NaAc buffer (0.1 M, pH 5.0) containing HEA NWs (5 μg mL^-1^) reacting with various concentrations of TMB (0.1 to 0.6 mM) and a fixed concentration of H_2_O_2_ (10 mM) or varying the concentrations of H_2_O_2_ (1.0 to 80 mM) while keeping the concentration of TMB (1.0 mM). All the above reactions were performed by monitoring the oxidation of TMB at 652 nm every 7.5 s. Afterward, the catalytic kinetic parameters (*K_m_* and *v*_max_) were obtained from Lineweaver-Burk plots of the double inverse of the Michaelis-Menten equation *v* = *v_max_* × [S]/(*K_m_* + [S]), where *v* is the initial velocity, *v*_max_ is the maximal reaction rate, [S] is the concentration of substrate, and *K_m_* is the Michaelis constant.

**Assessment of Specific Activity.** Typically, a series of amounts (0.2 to 1 μg) of HEA NWs were added into pH 5.0 HAc-NaAc buffer (0.1 M) in the presence of TMB (0.5 mg mL^-1^) and H_2_O_2_ (1 M). The above mixtures were measured at 652 nm every 10 s for up to 400 s and the initial rate of change in absorbance was calculated from ∆A/∆t. Further, the catalytic activity of the nanozyme was obtained based on the values of ∆A/∆t and plotted against the nanozyme weight to determine the specific activity.

**Determination of ESR.** Electron spin resonance (ESR) spectroscopy was applied to identify and quantify free radicals including •O_2_^-^, •OH, and ^1^O_2_. In brief, ESR testing was performed in pH 5.0 HAc-NaAc buffer (0.1 M) containing H_2_O_2_, HEA NWs, and tarp agents (5,5-dimethyl-1-pyrroline-oxide (DMPO) for •O_2_^-^ and •OH, 2,2,6,6-tetramethylpiperidine (TEMP) for ^1^O_2_) after incubating for 5 min at room temperature.

**Density Functional Theory Calculations.** To demonstrate the performance of PdMoPtCoNi HEA NWs, the Vienna Ab Initio Package (VASP)^[43]^ was introduced to conduct all DFT calculations with the Generalized Gradient Approximation (GGA) and Perdew-Burke-Ernzerhof (PBE)^[44]^ functionals in this work. The projection-enhanced wave (PAW) potential^[45]^ was employed to depict the ionic cores and valence electrons were brought into account using a plane-wave basis with a kinetic energy cut-off set to 450 eV. Partial occupancies of the Kohn-Sham orbitals were allowed with a width of 0.05 eV by using the Gaussian smearing method. When the energy change was < 10^-5^ eV, the electron energy was assumed to be self-consistent. When the change in force was < 0.03 eV Å^-1^, the geometric optimization was regarded as converged. Grimme's DFT-D3 method was utilized for the description of dispersion interactions.^[46]^ The Brillouin zone integral used the surface structures of Gamma-centered K point sampling for the PdMoPt and PdMoPtCoNi in a large size.

**Fabrication and Evaluation of** **the Portable Electronic Device.** A portable electronic device costing 25 USD was fabricated with three channels for glucose, *P. mirabilis*, and sarcosine colorimetric detection, respectively. The shell and multi-channel chamber of the device were designed by Autodesk123D Design (Autodesk Inc, San Francisco, USA) and printed by a 3D printer (Aurora Technology Co., ltd., Shenzhen, China) using polylactic acid (PLA). The chamber consisted of a sample holder for an 8-well ELISA plate strip as the cuvette with holes on either side for the LED (652 nm, 3 mm in diameter) and OS required. The main parts of the readout circuit included an ADS1115 16-bit ADC, an MCU (IoT chip ESP8266 with built-in WIFI), and an LCD 1602 display. Notably, this device was aimed to simplify the spectroscopy system and enable the monitoring of the light intensity of solutions contained in the plate strip easily. To evaluate the performance of the device in optical measurement, colored assay solutions were prepared by mixing HEA NWs (at various concentrations), TMB (1.0 mM), and H_2_O_2_ (10 mM) in HAc-NaAc buffer (0.1 M, pH 5.0). To simplify data processing for the user, the subsequently obtained calibration curves for the three targets were imported into the device, so that the device could further report the target concentrations directly by E-mail via IoT technology.

**Colorimetric Detection of Glucose.** Glucose solutions at different concentrations (1 to 400 μM) mixed with GOX (40 μg mL^-1^), 1.0 mM TMB, and 5 μg mL^-1^ HEA NWs were dispersed in 0.1 M HAc-NaAc buffer (pH 5.0). After incubating at 37 ºC for 15 min, the prepared reaction mixture was subjected to measure absorption spectra and record absorption at 652 nm. The proposed protocol was also performed in glucose monitoring in artificial urine. The artificial urine sample was diluted with water at a volume ratio of 1:49 and dissolved varied amounts of glucose. Then, the mixed solutions were incubated with GOX and prepared for further glucose assay.

**Colorimetric Detection of Sarcosine.** A series of concentrations of sarcosine solutions (10 to 2500 μM) were incubated with SOX (25 U mL^-1^) under 37 ºC for 10 min. Afterward, the prepared mixture (1/10 of total reaction volume), TMB (1.0 mM), and HEA NWs (5 μg mL^-1^) were reacted in HAc-NaAc buffer (pH 5.0, 0.1 M) under room temperature for 20 min. After that, the UV spectra of the reaction solutions were recorded. To further evaluate the feasibility of the strategy above in urine detection, diluted urine samples with sarcosine were used for detection.

**Colorimetric Detection of Urease Activity.** Various concentrations of urease (PBS, pH 7.4) were mixed with urea (1.8 M) at 37 ºC for 10 min. Then, 1.0 mM TMB, 10 mM H_2_O_2_, 5 μg mL^-1^ HEA NWs, and the above solution were introduced to 0.1 M HAc-NaAc buffer (pH 5.0). After the reaction under room temperature for 10 min, the absorbance values of the reaction mixtures at 652 nm were measured.

**Colorimetric Detection of *P. mirabilis*.** *P. mirabilis* (ATCC 12453) were cultured on LB nutrient agar plates at 37 ºC for 24 h. Subsequently, a single colony of *P. mirabilis* was placed into 25 mL of TSB and incubated at 37 ºC with shaking at 120 rpm for 12 h. The bacteria solution was obtained by centrifuging at 6000 rpm (4 ºC, 3 min) and resuspended in sterile PBS (pH 7.4). Urea (2 M) was added to the *P. mirabilis* solutions with different concentrations (10^1^ to 10^8^ CFU mL^-1^) and co-incubated at 37 ºC for 50 min. Afterward, the prepared mixture was transferred into pH 5.0 HAc-NaAc buffer (0.1 M) containing HEA NWs (5 μg mL^-1^), TMB (1.0 mM), H_2_O_2_ (10 mM). The absorbances of all systems at 652 nm were determined after reacting for 10 min at room temperature. Moreover, the same strategy was applied to test the *P. mirabilis* in artificial urine samples.

**Selectivity Analysis for Colorimetric Assay.** Several possible interfering substances were introduced to the HEA NWs/TMB chromogenic system to verify the selectivity of the colorimetric detection. The absorption of all testing groups was recorded at 652 nm after incubation for 5 min at room temperature.

**Selectivity Test for *P. mirabilis* Detection.** To assess the selectivity of *P. mirabilis* monitoring, *E. coli*, *S. aureus*, and *L. monocytogenes* were used for colorimetric assay. Briefly, the bacteria were cultured in liquid LB broth in an incubator shaker (37 ºC, 120 rpm) for 12 h. After being washed with sterile PBS (pH 7.4), the redispersed bacteria solutions were conducted for analysis with all concentrations of 10^8^ CFU mL^-1^.

**Statistical Analysis.** All data were given as mean ± standard deviation (SD) and the Student’s t-test was applied to analyze the difference between the mean values of the two groups using Minitab 19.0 software with **p* < 0.05 and ***p* < 0.01.

**Table S1.** Comparison of the kinetic parameters of PdMoPtCoNi HEzymes with PdMoPt, PdPtCo, PdPtNi, PdMoPtCo, PdMoPtNi, HRP, and previously reported nanozymes.

| Catalyst | Michael's constant | | Maximum reaction rate | | Reference |
| --- | --- | --- | --- | --- | --- |
|  | *K_m_* (mM) | | *v*_max_ (10^-8^ M•s^-1^) | |  |
|  | TMB | H_2_O_2_ | TMB | H_2_O_2_ |  |
| HRP | 0.43 | 3.70 | 10.00 | 8.71 | [47] |
| PtCu | 0.39 | 0.33 | 5.54 | 1.14 | [48] |
| PdBi | 0.23 | 590 | 43 | 22 | [49] |
| AuPtCo | 0.137 | 2.593 | 1.798 | 10.16 | [50] |
| Cu_2_O/Au–Pt@MOF@F127 | - | 22.4 | - | 4.05 | [51] |
| Ir-N_5_ SA | - | 2.43 | - | 9.23 | [52] |
| PEGylated Pd SAzyme | - | 1.79 | - | 15.1 | [53] |
| Ti_3_C_2_T*_x_*-Pt-PEG | - | 34.91 | - | 7.22 | [54] |
| PdMoPt | 0.62 | 14.68 | 8.92 | 12.43 | This work |
| PdPtCo | 0.31 | 2.75 | 12.26 | 22.80 |  |
| PdPtNi | 0.43 | 11.92 | 7.06 | 12.08 |  |
| PdMoPtCo | 0.22 | 2.04 | 10.72 | 23.15 |  |
| PdMoPtNi | 0.56 | 13.96 | 9.12 | 12.35 |  |
| PdMoPtCoNi HEzymes | 0.073 | 0.73 | 12.27 | 29.02 |  |

**Table S2.** Comparison of the analytical performance of the proposed colorimetric strategy with previously reported assays.

| Target | Catalyst | Limit of detection (LOD) | Reference |
| --- | --- | --- | --- |
| Glucose | Ag@Fabric | 80 μM | [55] |
|  | CoFe-NG | 190 μM | [56] |
|  | PAni-NPs | 540 μM | [57] |
|  | PdMoPtCoNi HEzymes | 0.41 μM | This work |
| *P. mirabilis* | Gold nanoparticles | 10^1^ Cells mL^-1^ | [58] |
|  | β-CD/AuBi | 4 CFU mL^-1^ | [40] |
|  | PdMoPtCoNi HEzymes | 1.81 CFU mL^-1^ | This work |
| Sarcosine | MnO_2_ NSs | 0.36 μM | [59] |
|  | Fe_3_O_4_@SiO_2_@NiCo_2_S_4_ | 0.42 μM | [60] |
|  | Pt-Fe_3_O_4_@C | 0.43 μM | [61] |
|  | PdMoPtCoNi HEzymes | 0.32 μM | This work |

**Table S3.** Detection of glucose, *P. mirabilis*, and sarcosine in urine samples (n=3).

| Target | Spiked concentration | Measured concentration | Recovery rate (%) | RSD  (%) |
| --- | --- | --- | --- | --- |
| Glucose | 25 μM | 25.98 μM | 103.92 | 9.09 |
|  | 100 μM | 117.34 μM | 117.34 | 3.08 |
|  | 200 μM | 203.18 μM | 101.59 | 6.98 |
| *P. mirabilis* | 10^2^ CFU mL^-1^ | 10^1.95^ CFU mL^-1^ | 89.58 | 8.23 |
|  | 10^5^ CFU mL^-1^ | 10^5.05^ CFU mL^-1^ | 113.29 | 7.31 |
|  | 10^7^ CFU mL^-1^ | 10^6.92^ CFU mL^-1^ | 82.78 | 8.64 |
| Sarcosine | 30 μM | 25.71 μM | 85.69 | 8.18 |
|  | 150 μM | 142.84 μM | 95.23 | 7.10 |
|  | 250 μM | 259.17 μM | 103.67 | 9.71 |


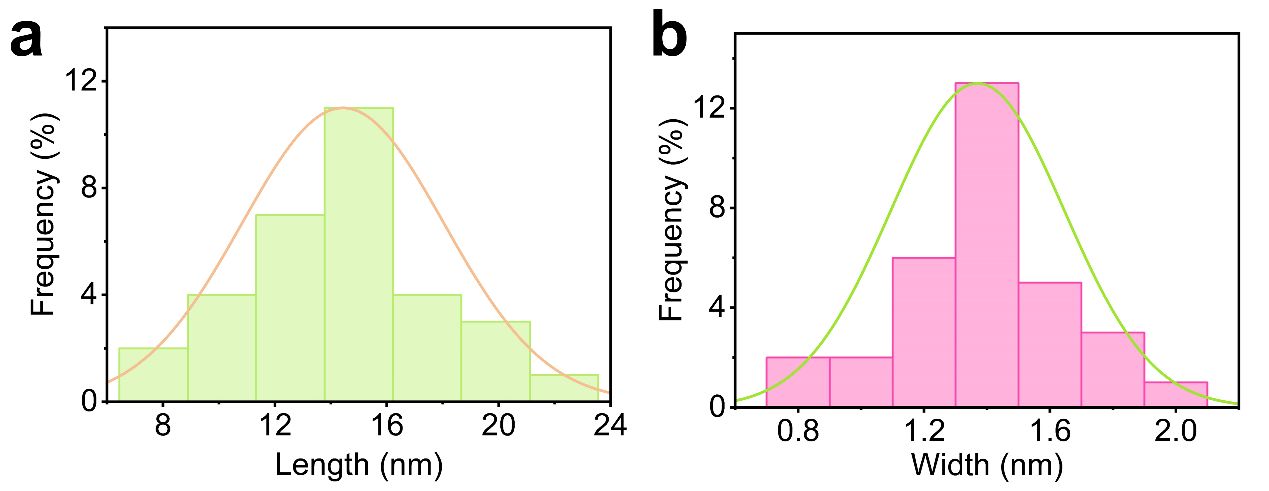


**Figure S1.** Size distribution of the HEA NWs.


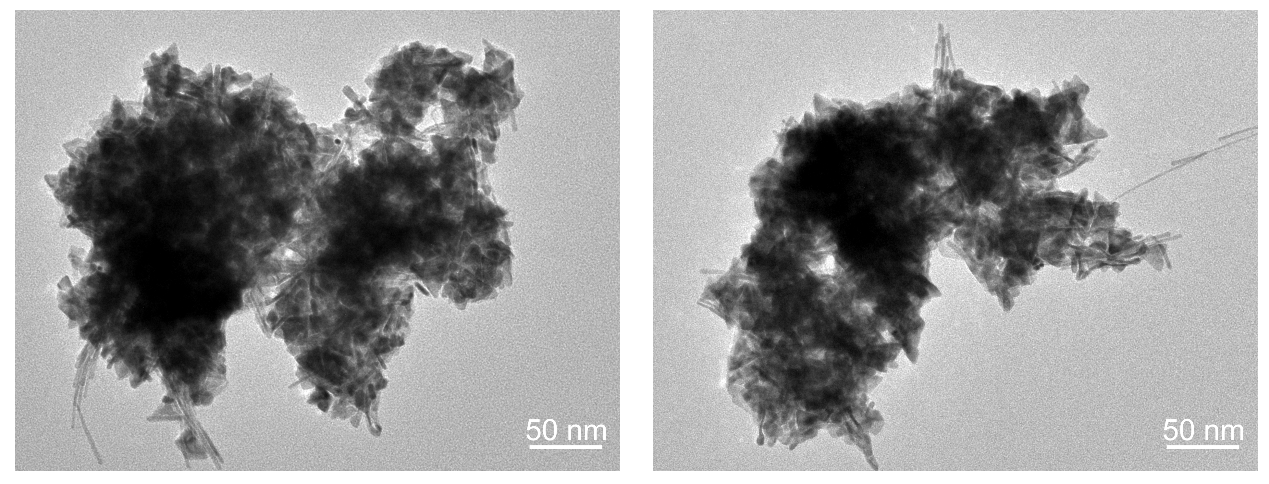


**Figure S2.** TEM images of the products synthesized with Mo(CO)_6_ (20 mg), glucose (10 mg), and CTAB (128 mg).


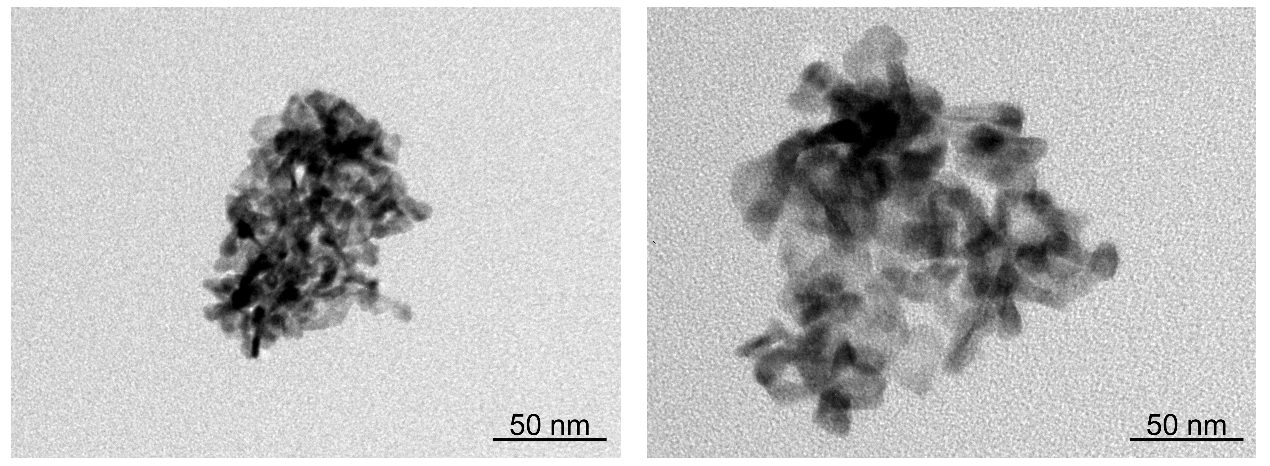


**Figure S3.** TEM images of the products synthesized with Mo(CO)_6_ (20 mg), glucose (60 mg), and CTAB (128 mg).

**
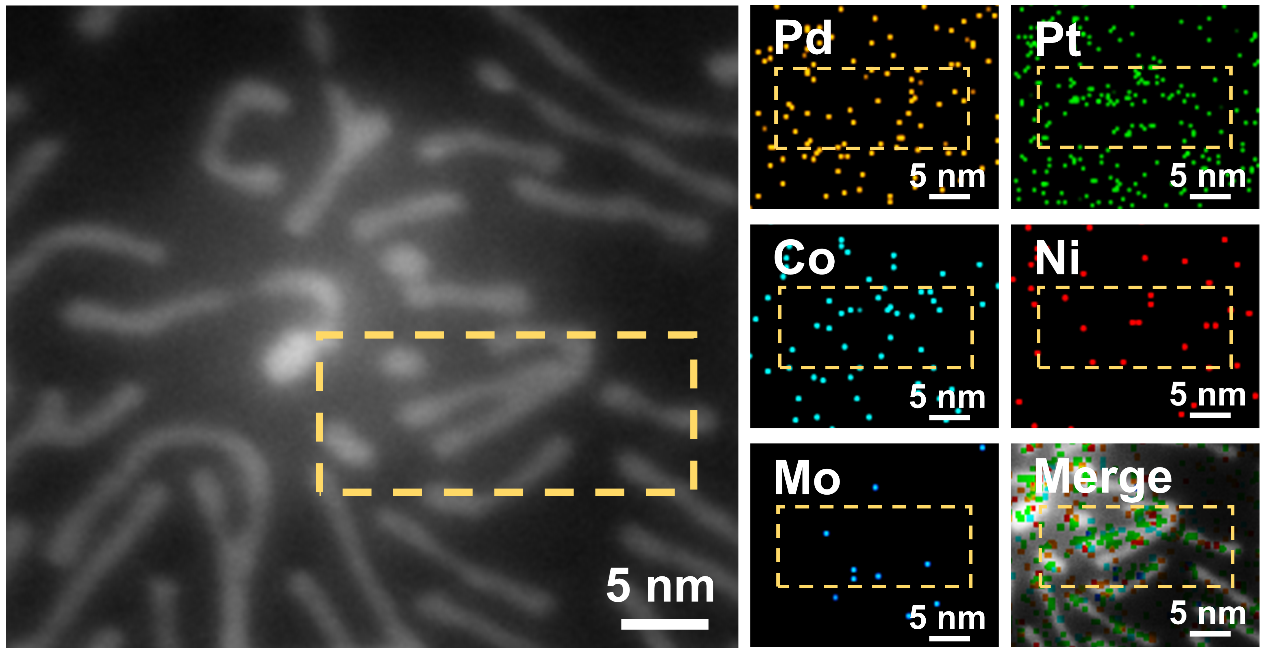
**

**Figure S4.** EDS Mapping characterization of the HEA NWs.


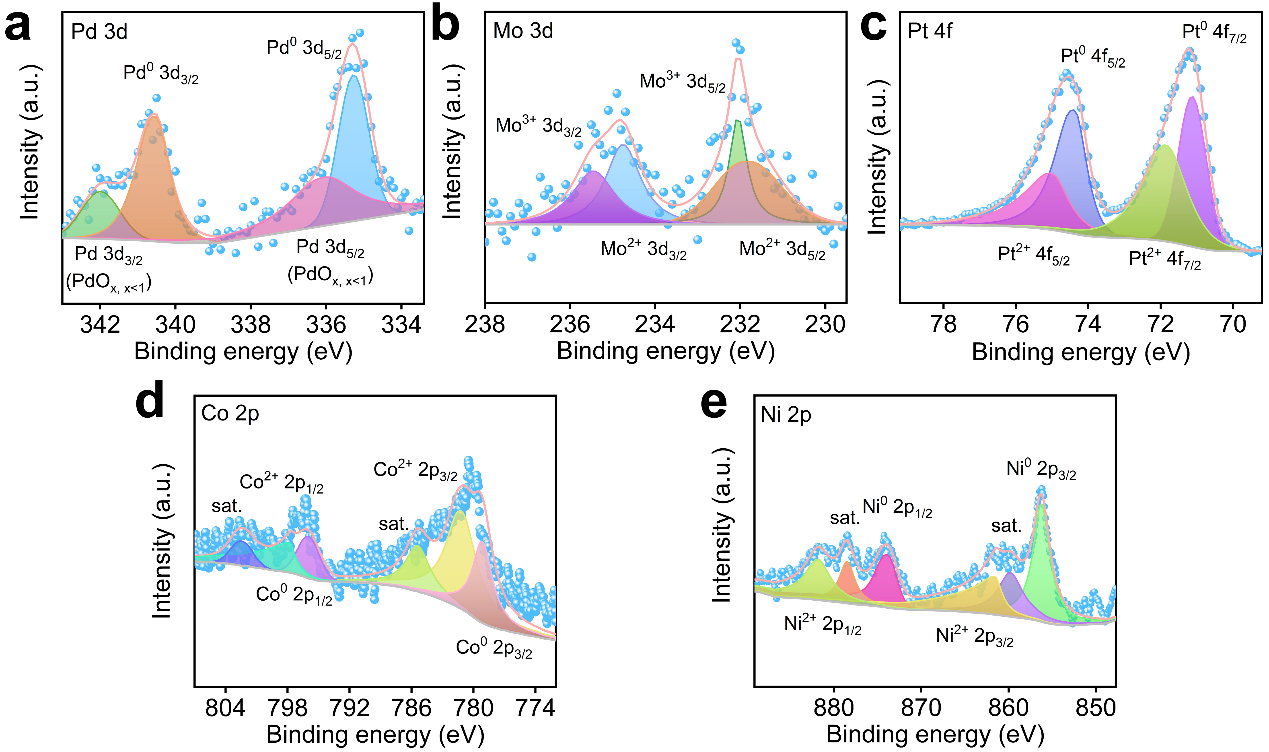


**Figure S5.** XPS spectra of (a) Pd 3d, (b) Mo 3d, (c) Pt 4f, (d) Co 2p, and (e) Ni 2p for the HEA NWs.


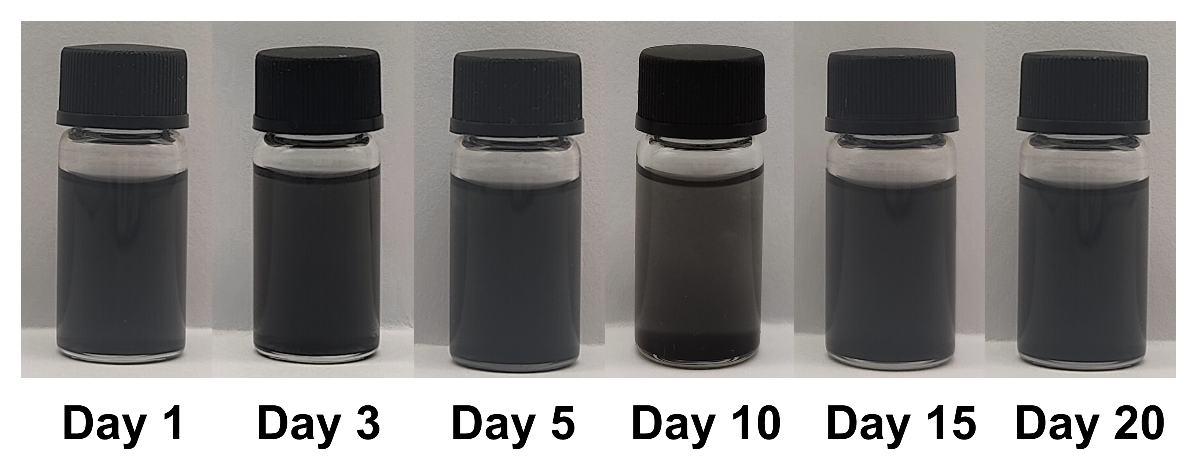


**Figure S6.** Images of the HEA NWs aqueous solutions at room temperature with various storage days.


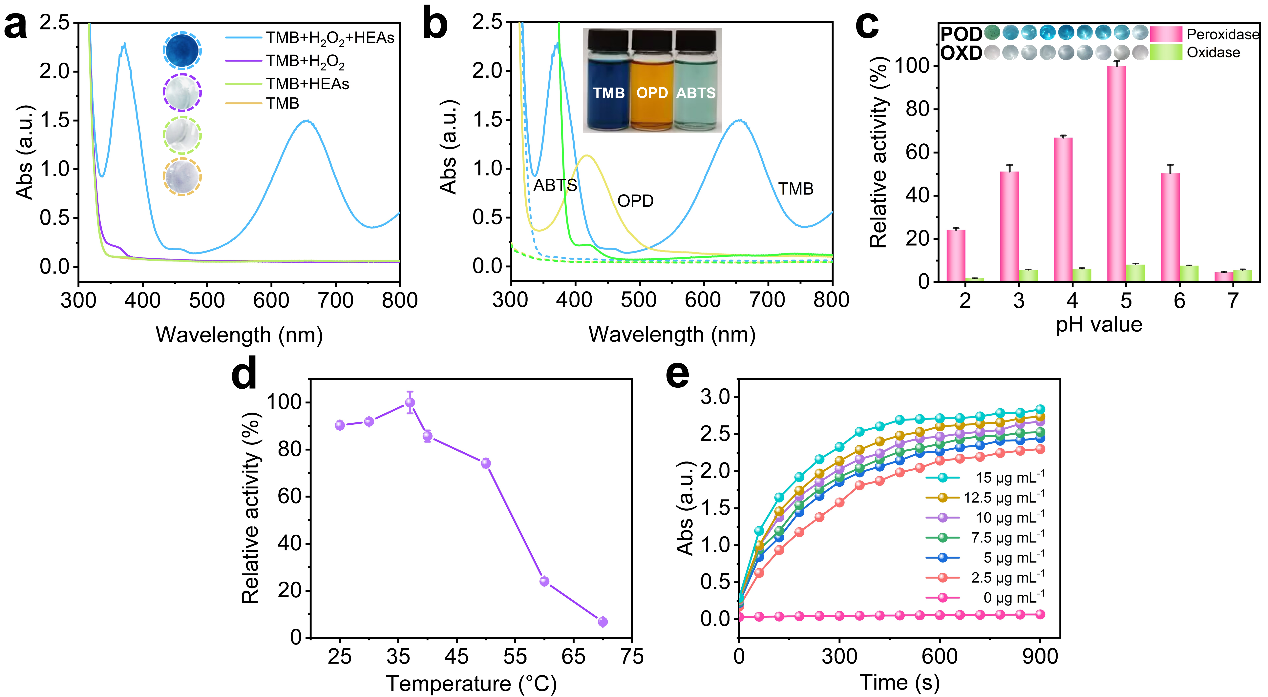


**Figure S7.** The POD-like activity of the HEA NWs. (a) The UV-vis spectra of TMB in different systems. (b) UV-vis absorption spectra of HEA NWs-TMB, HEA NWs-OPD, and HEA NWs-ABTS. (c) The POD- and OXD-like activity of the HEA NWs under various pH values. The data are presented as mean ± SD (n = 3 independent samples). (d) The activity of the HEA NWs under various temperatures. The data are presented as mean ± SD (n = 3 independent samples). (e) Temporal changes of the absorbance at 652 nm for oxidation of different concentrations of the HEA NWs.

**
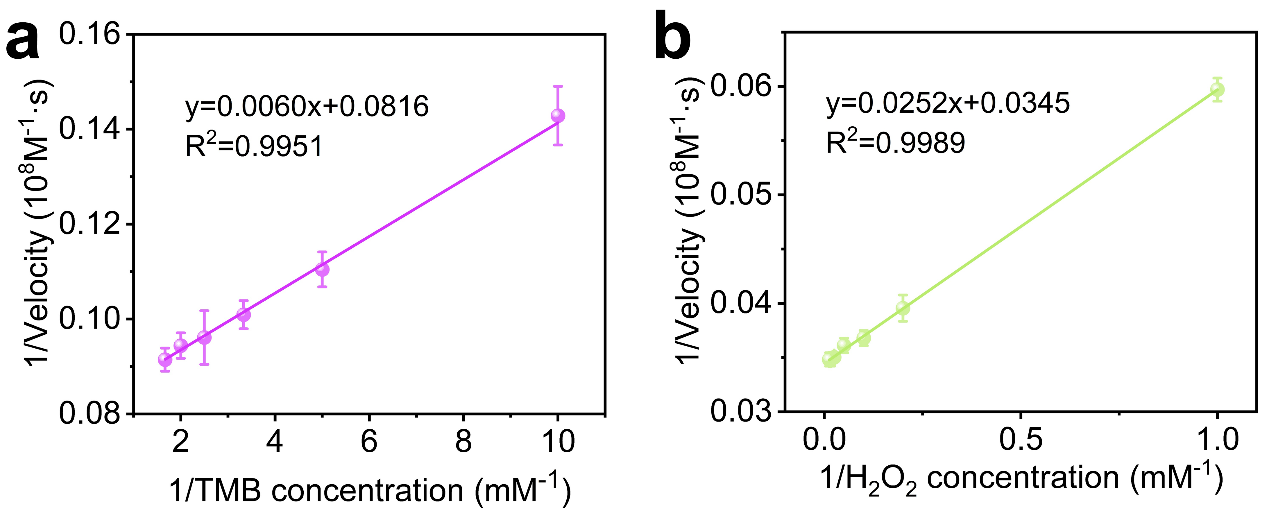
**

**Figure S8.** Lineweaver-Burk plots of the HEA NWs with (a) TMB and (b) H_2_O_2_ in HAc-NaAc buffer (0.1 M, pH 5.0) at room temperature. All data are presented as mean ± SD (n = 3 independent samples).


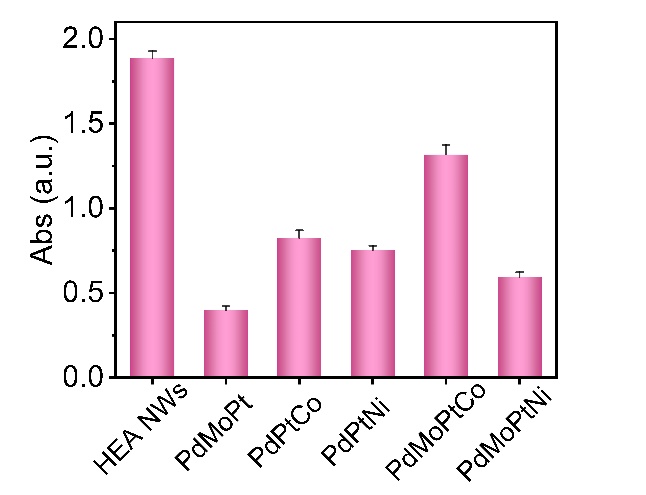


**Figure S9.** Comparison of the POD-like activity. All data are presented as mean ± SD (n = 3 independent samples).


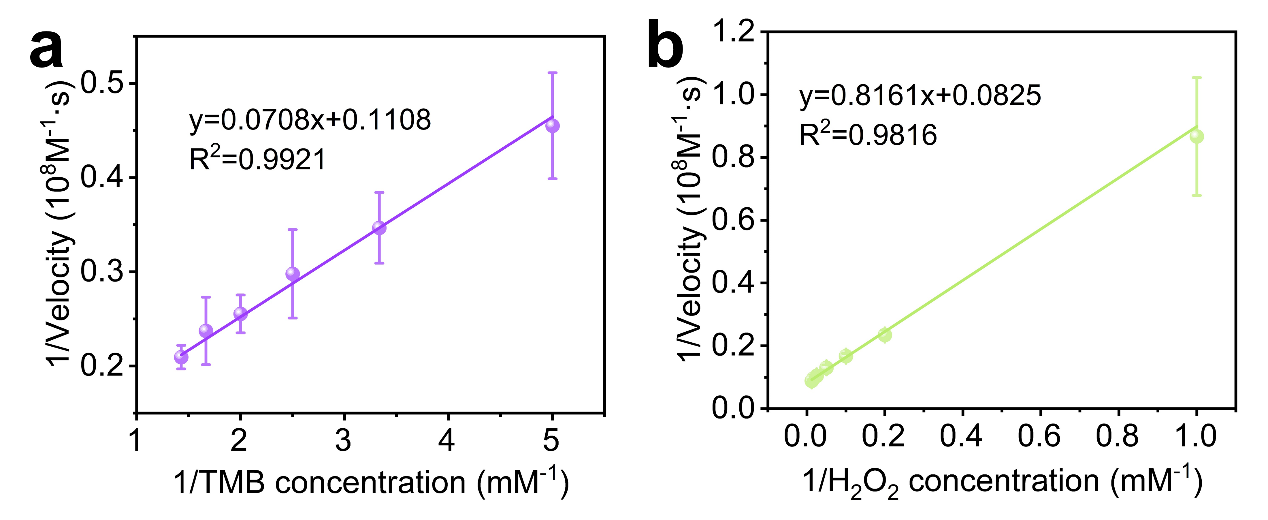


**Figure S10.** Lineweaver-Burk double reciprocal curve plots of PdMoPt with (a) TMB and (b) H_2_O_2_ in HAc-NaAc buffer (0.1 M, pH 5.0) at room temperature. All data are presented as mean ± SD (n = 3 independent samples).


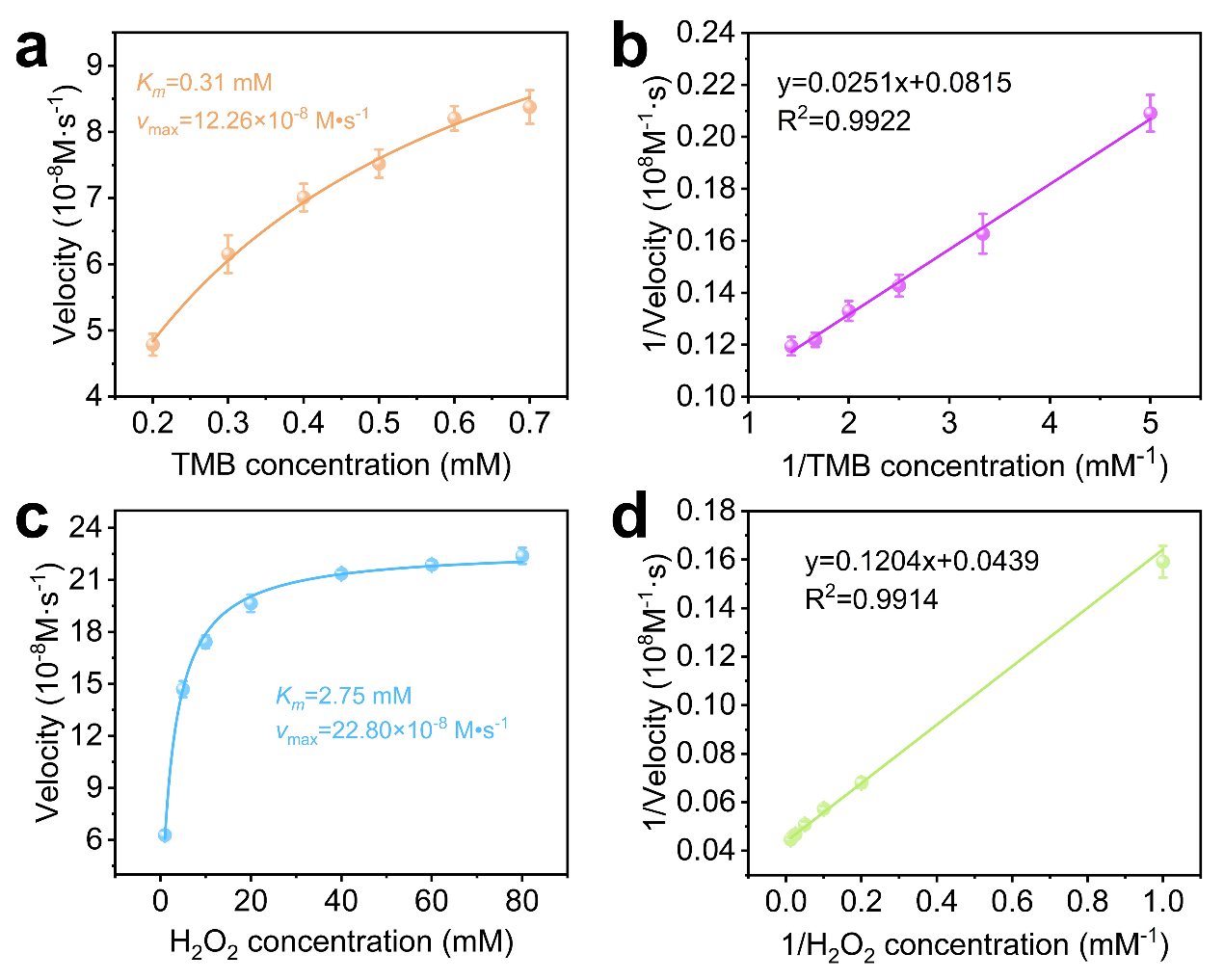


**Figure S11.** Enzyme kinetic performance of the PdPtCo. All data are presented as mean ± SD (n = 3 independent samples).


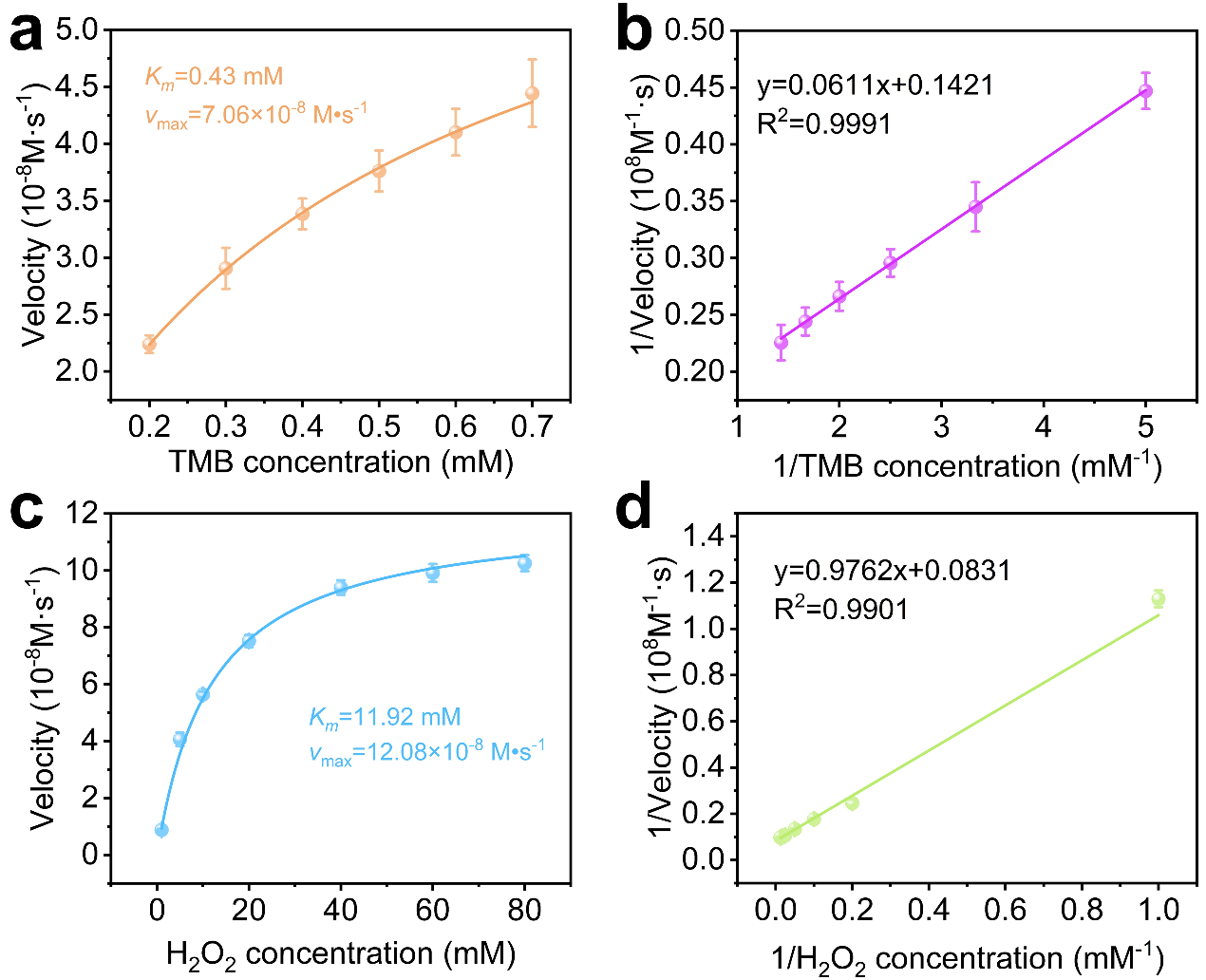


**Figure S12.** Enzyme kinetic performance of the PdPtNi. All data are presented as mean ± SD (n = 3 independent samples).


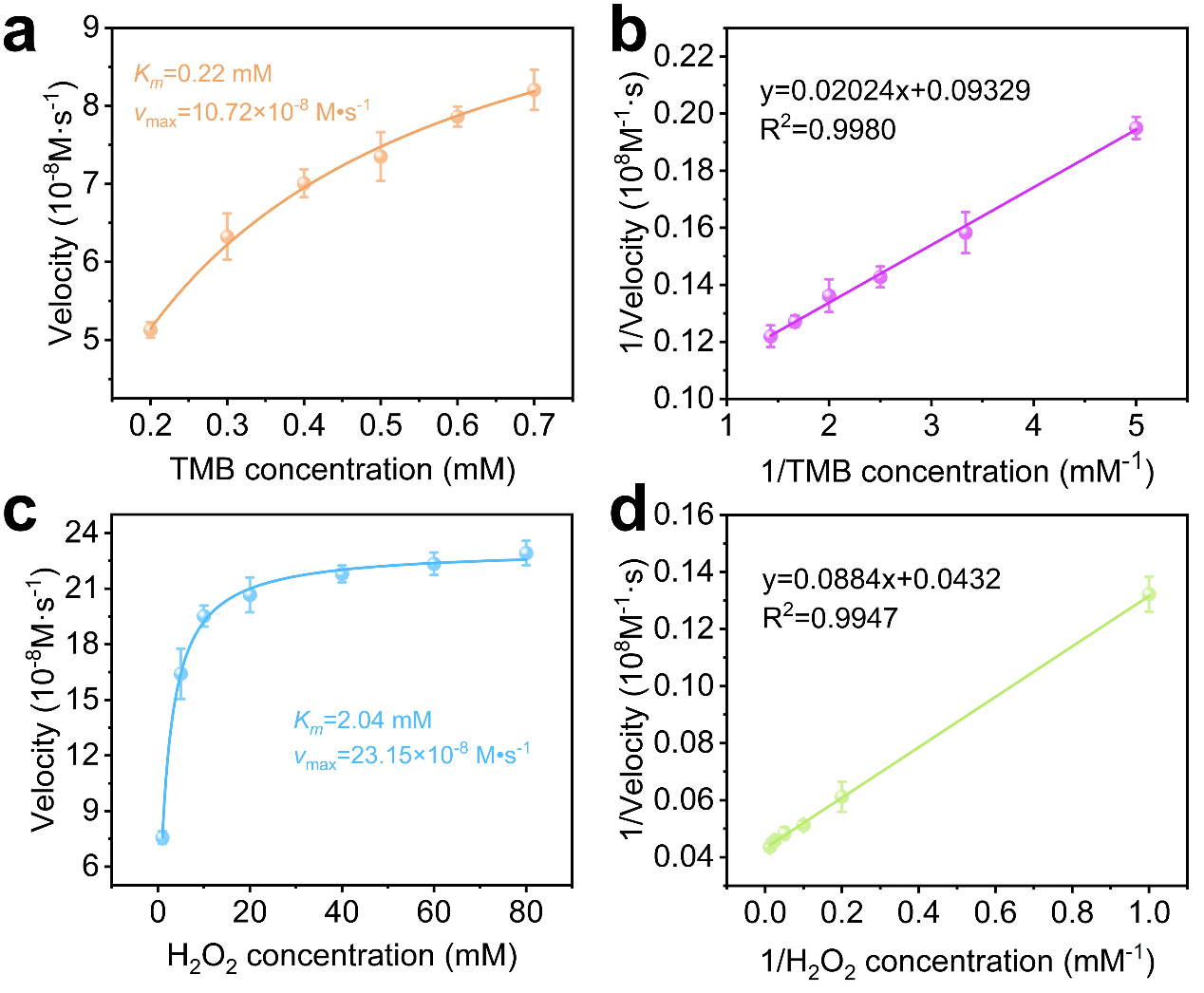


**Figure S13.** Enzyme kinetic performance of the PdMoPtCo. All data are presented as mean ± SD (n = 3 independent samples).


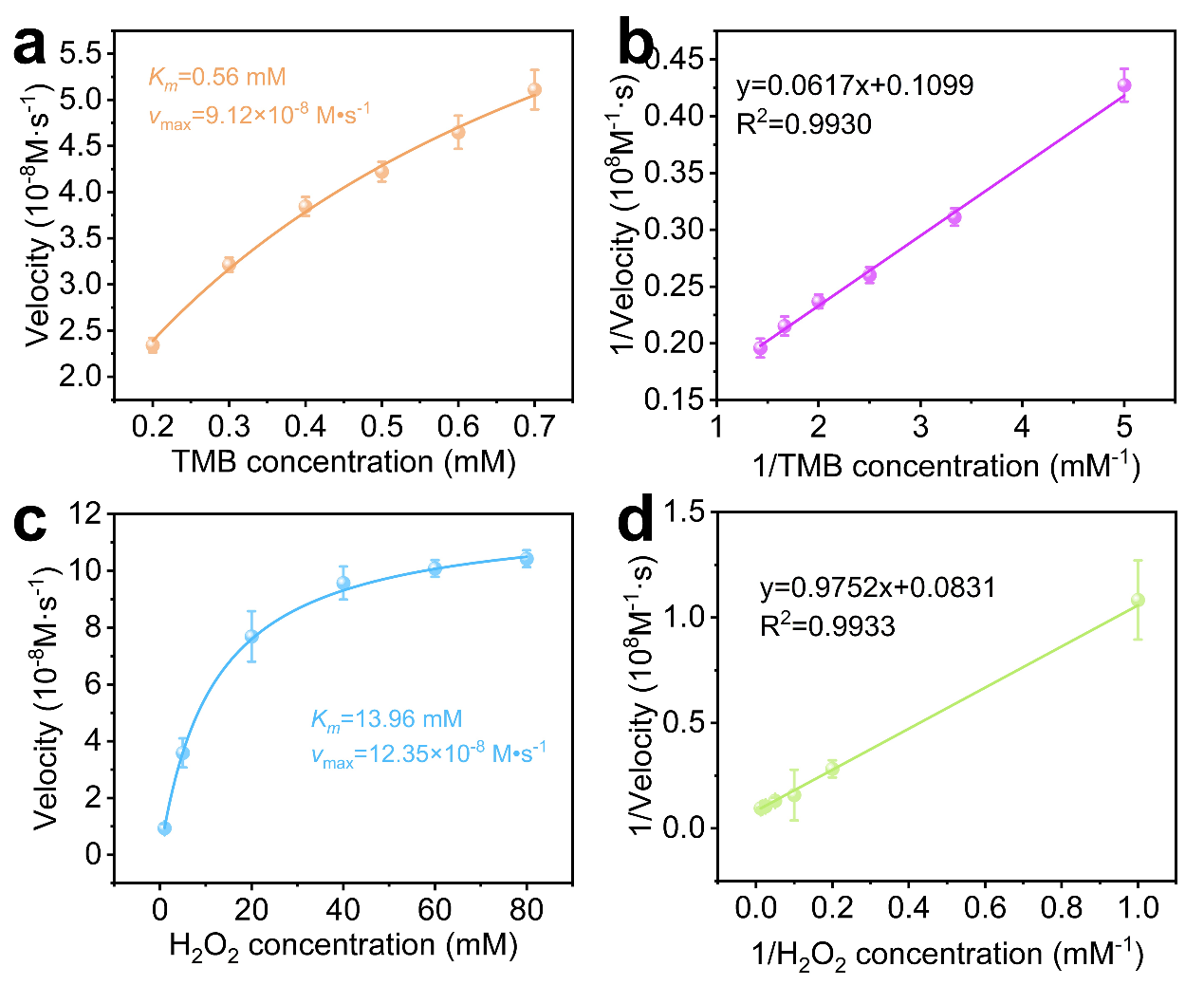


**Figure S14.** Enzyme kinetic performance of the PdMoPtNi. All data are presented as mean ± SD (n = 3 independent samples).


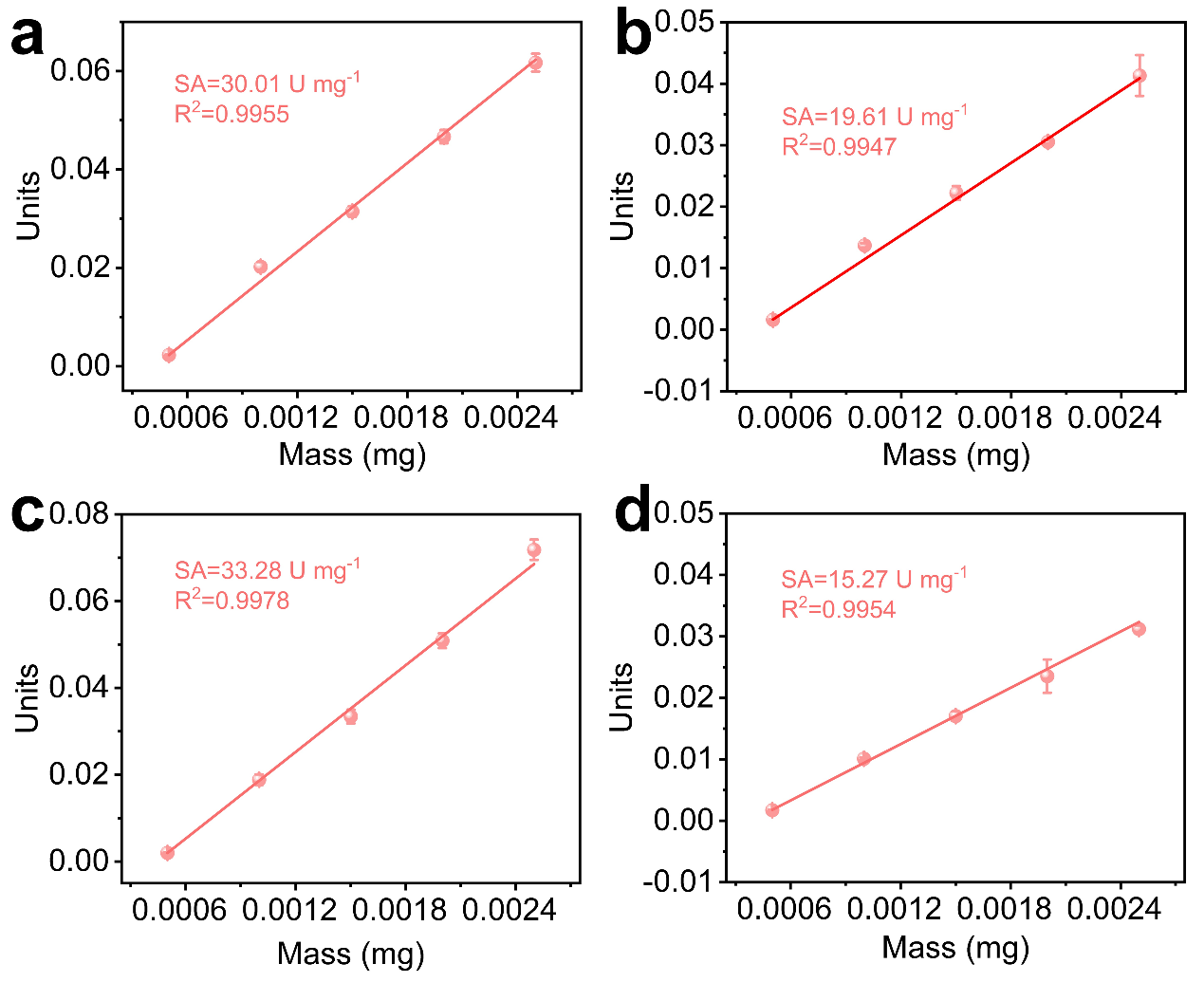


**Figure S15.** Specific activity of the PdPtCo, PdPtNi, PdMoPtCo, and PdMoPtNi. All data are presented as mean ± SD (n = 3 independent samples).


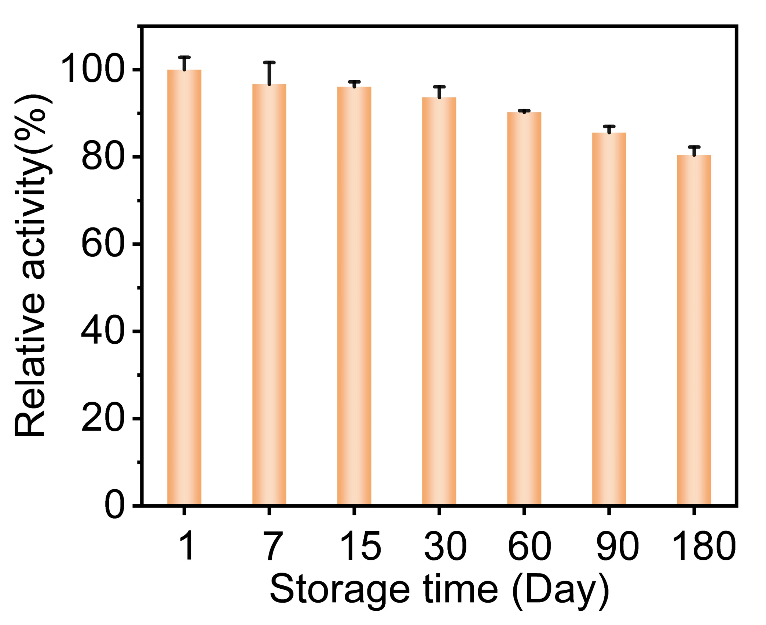


**Figure S16.** Investigation of the POD-like activity of HEA NWs at room temperature in different storage times. All data are presented as mean ± SD (n = 3 independent samples).


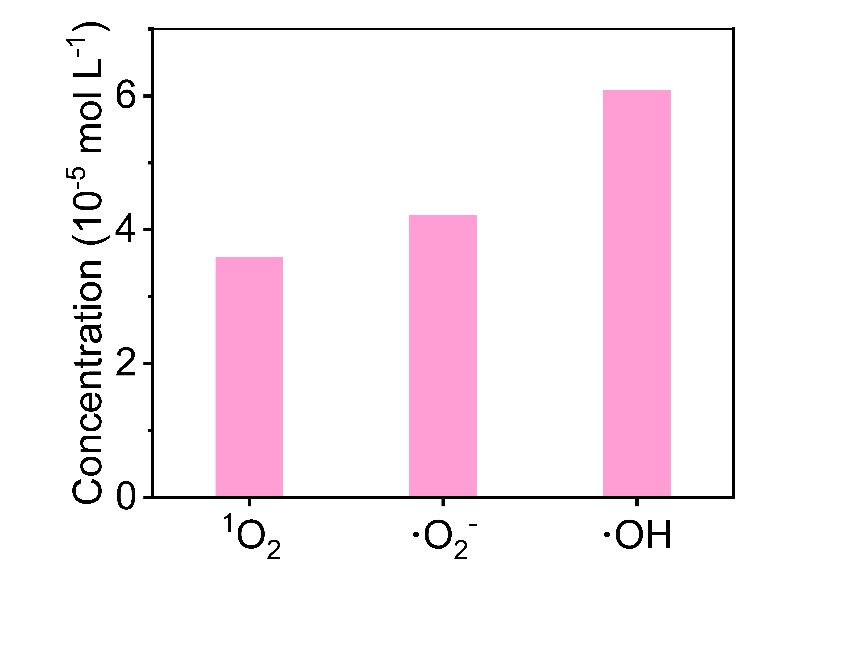


**Figure S17.** Concentration histogram of·OH, ·O_2_^-^ and ^1^O_2_.


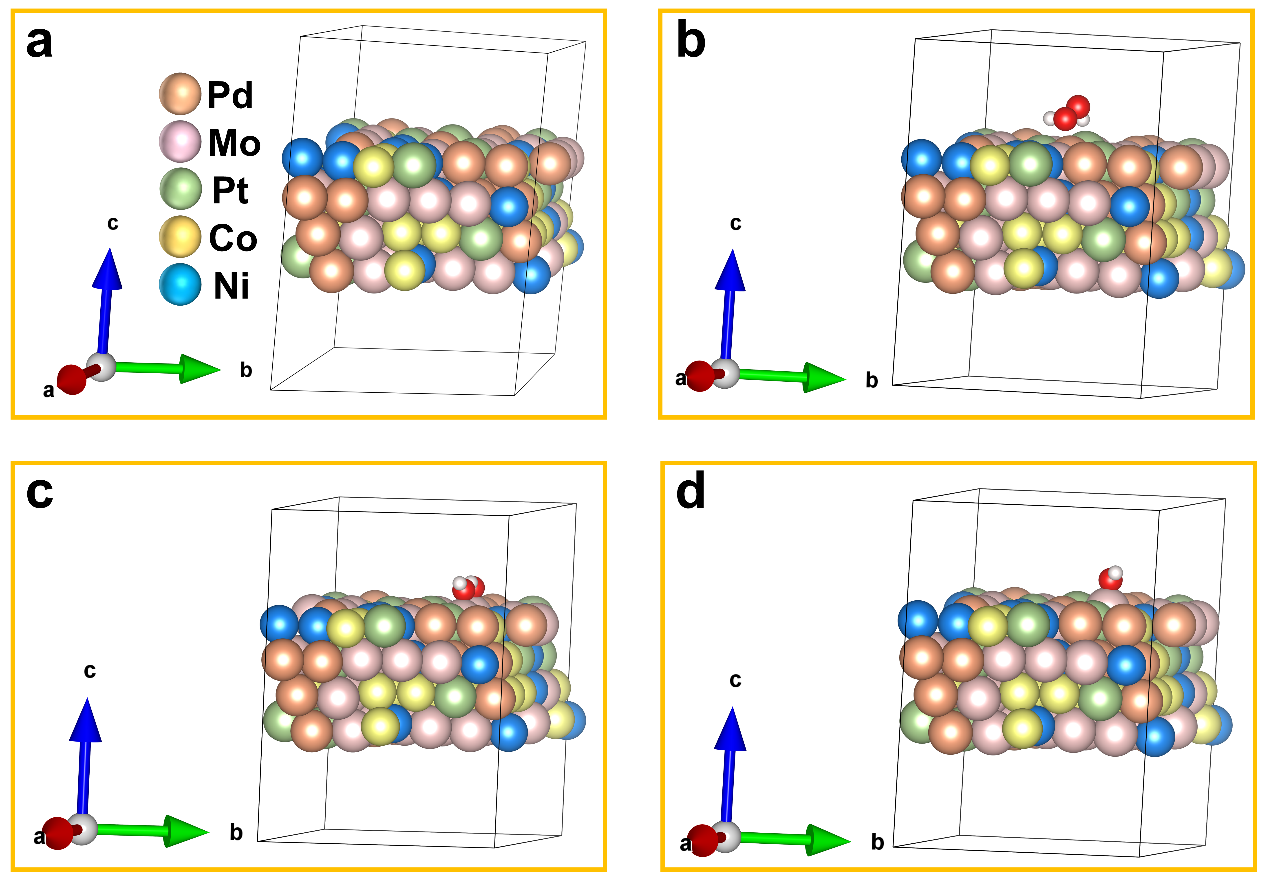


**Figure S18.** Construction of the HEA NWs model. Model of (a) HEAs, (b) H_2_O_2_ adsorption on HEAs surfaces, (c) two OH* on HEAs surfaces, and (d) one OH* on HEAs surfaces.


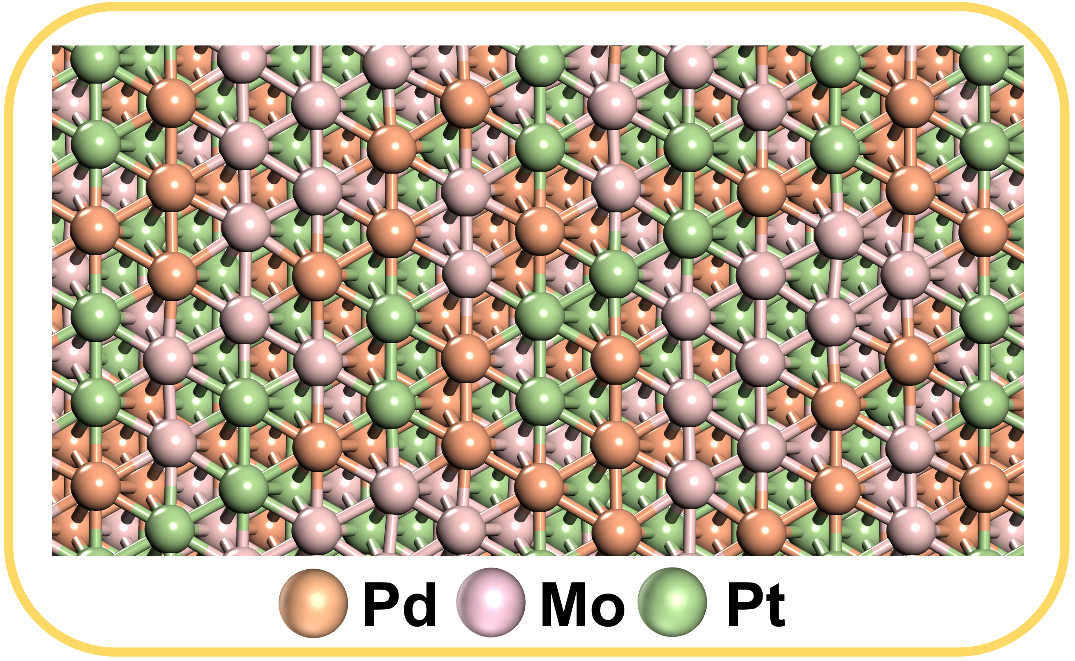


**Figure S19.** Top view of the optimized geometry of PdMoPt.


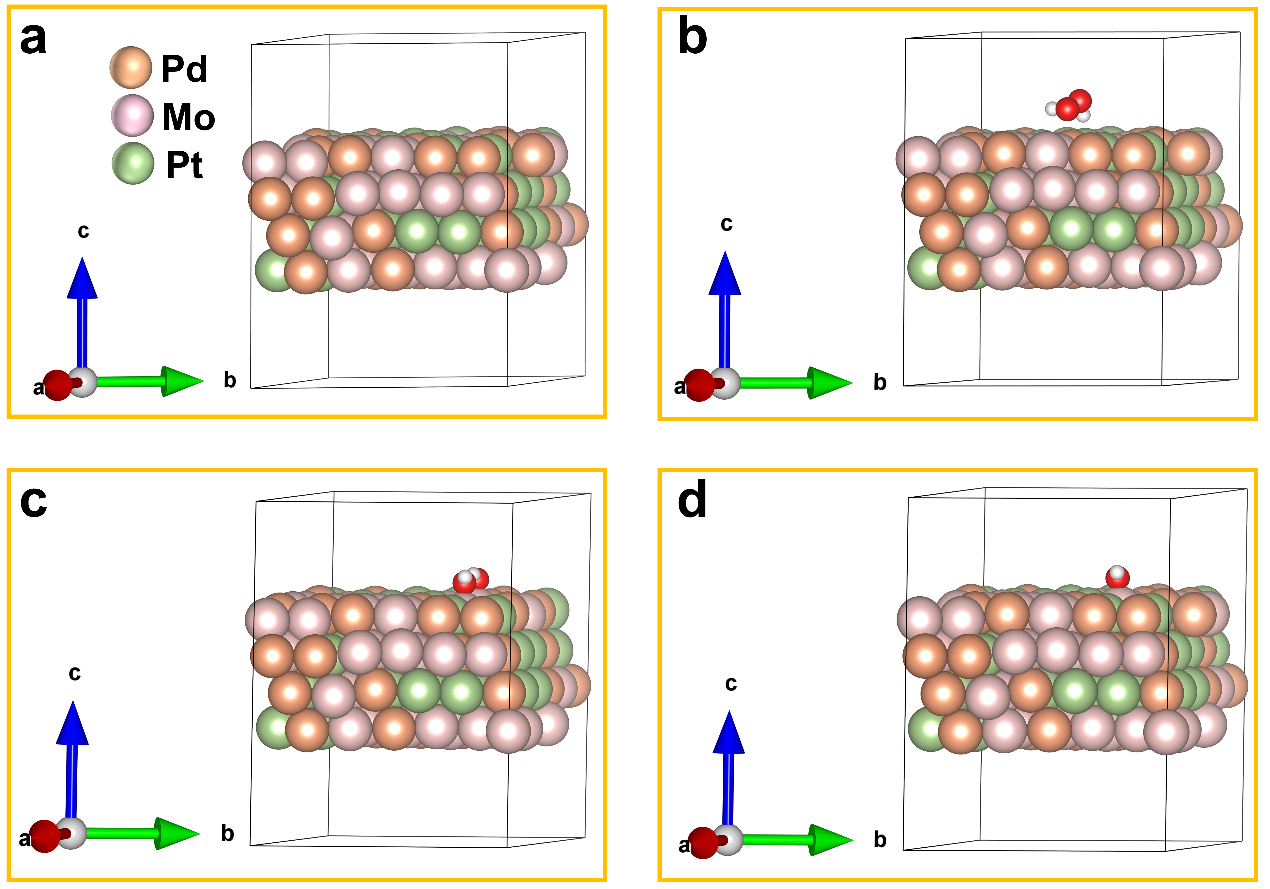


**Figure S20.** Construction of PdMoPt model. Model of (a) PdMoPt, (b) H_2_O_2_ adsorption on PdMoPt surfaces, (c) two OH* on PdMoPt surfaces, and (d) one OH* on PdMoPt surfaces.


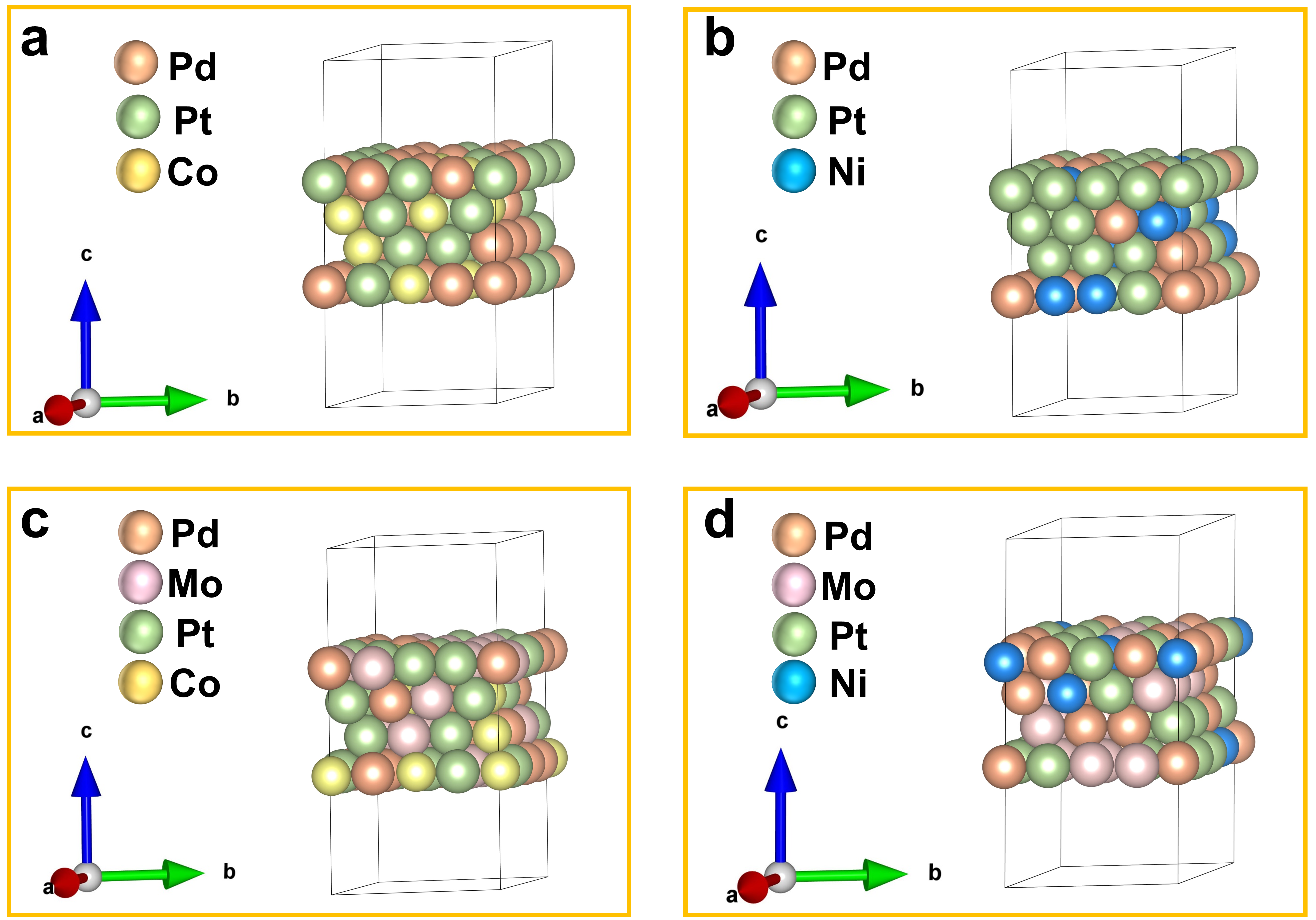


**Figure S21.** Construction of PdPtCo, PdPtNi, PdMoPtCo, and PdMoPtNi model.

**
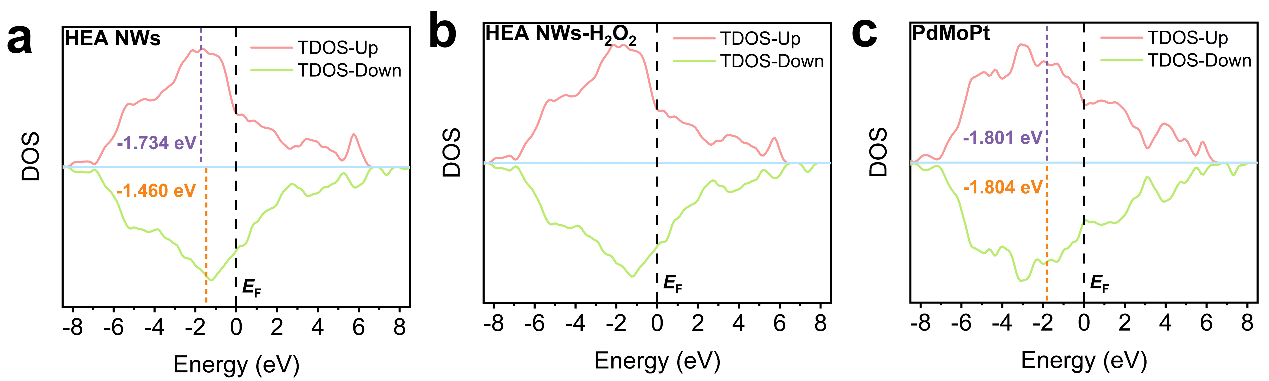
**

**Figure S22.** TDOS of (a) HEA NWs, (b) HEA NWs with H_2_O_2_ adsorbed on the surface, (c) PdMoPt.


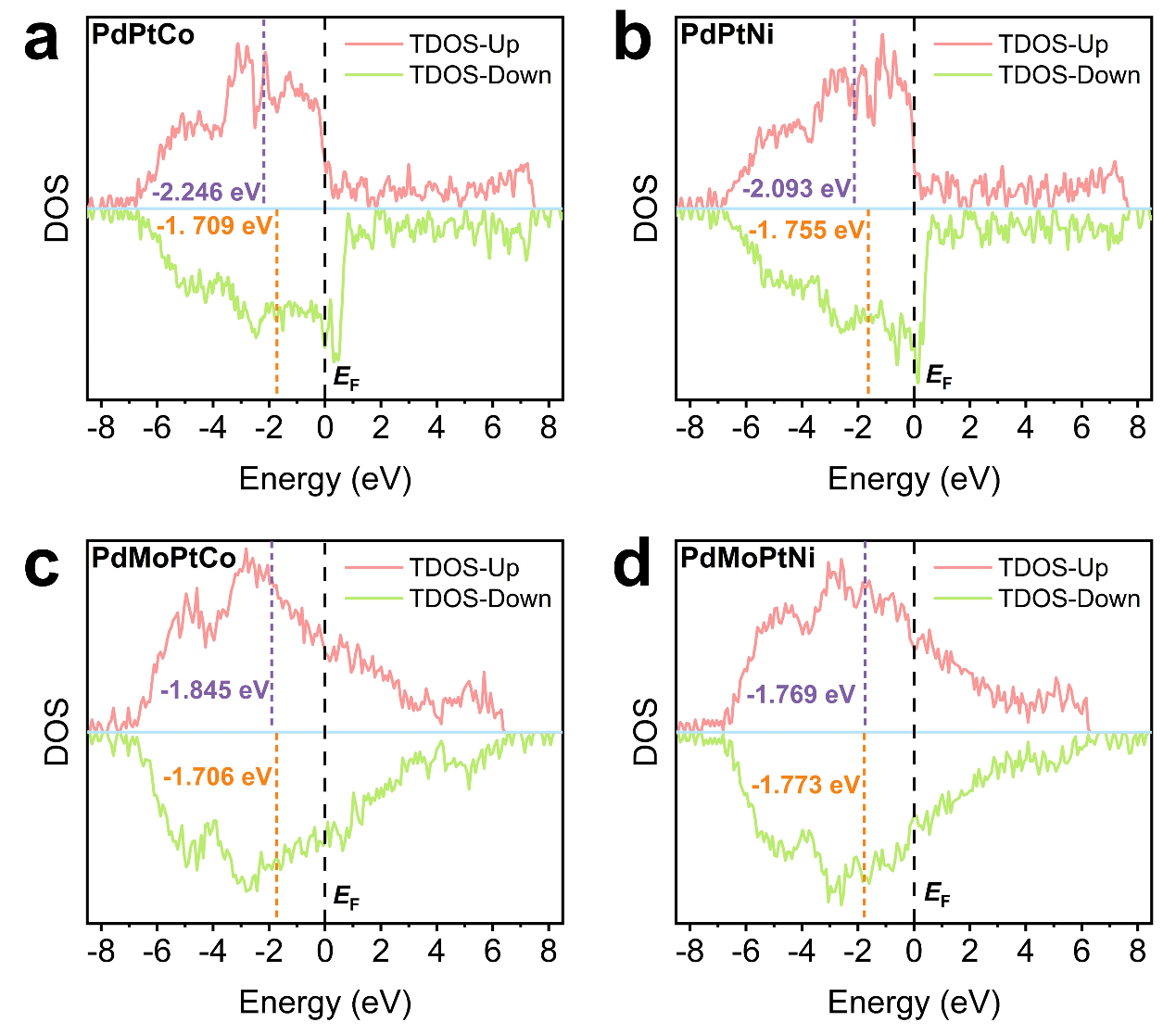
**Figure S23.** TDOS of (a) PdPtCo, (b) PdPtNi, (c)PdMoPtCo, and (d)PdMoPtNi.


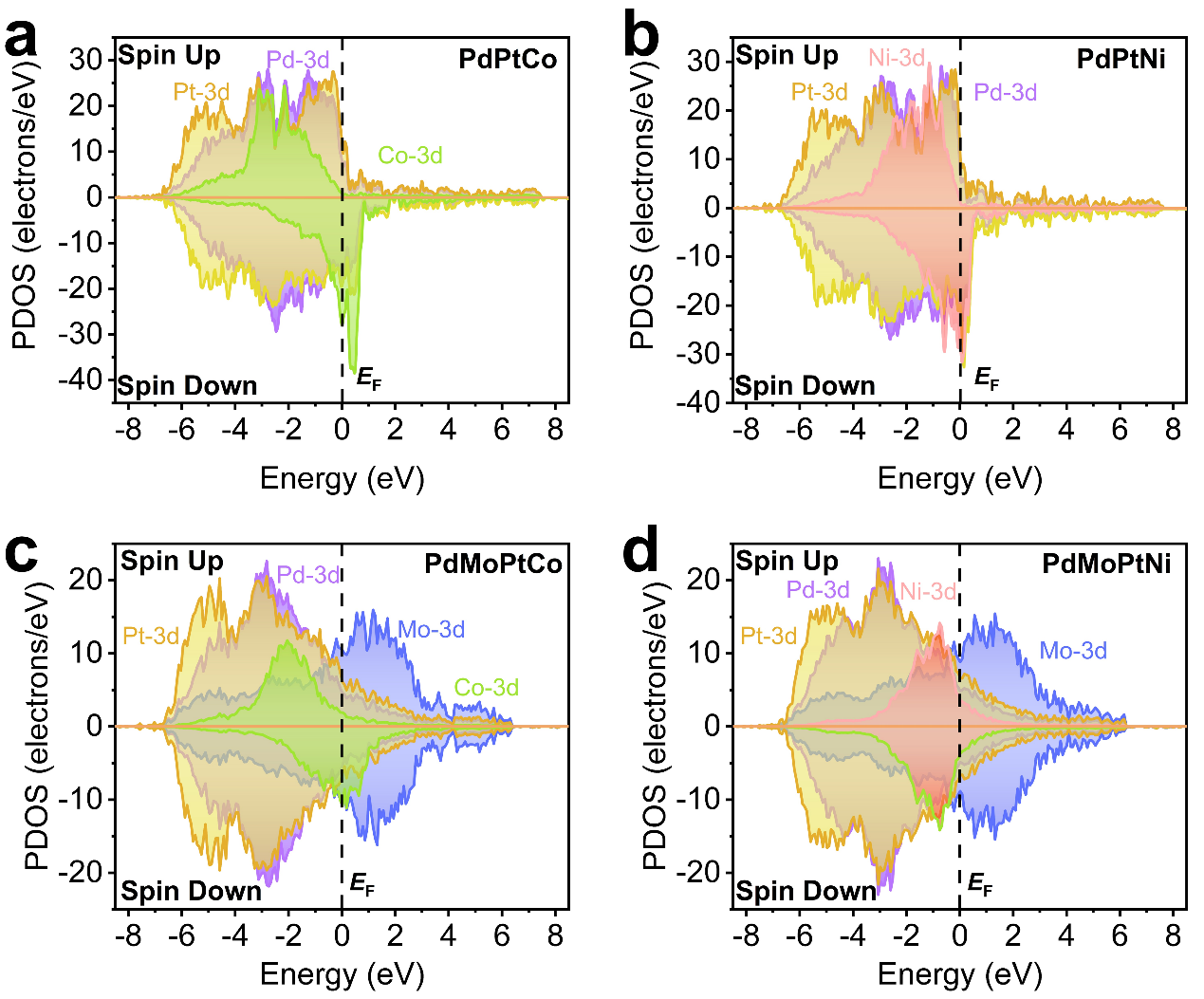
**Figure S24.** PDOS of (a) PdPtCo, (b) PdPtNi, (c)PdMoPtCo, and (d)PdMoPtNi.


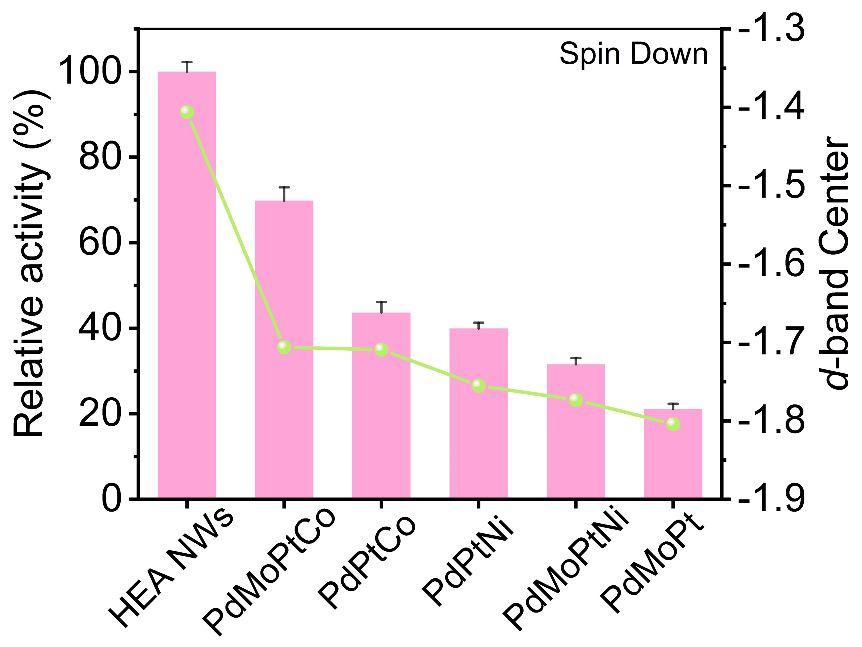


**Figure S25.** Comparison of *d*-band center and enzymatic activity.

**
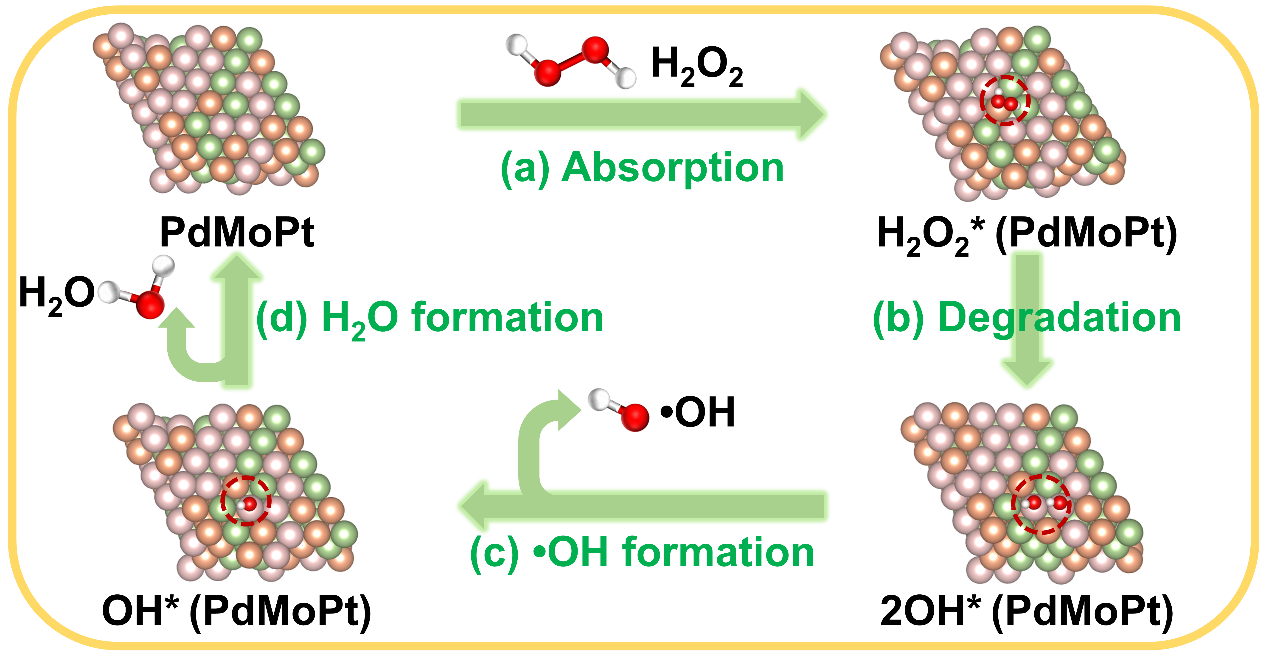
**

**Figure S26.** Proposed reaction process on PdMoPt.

**
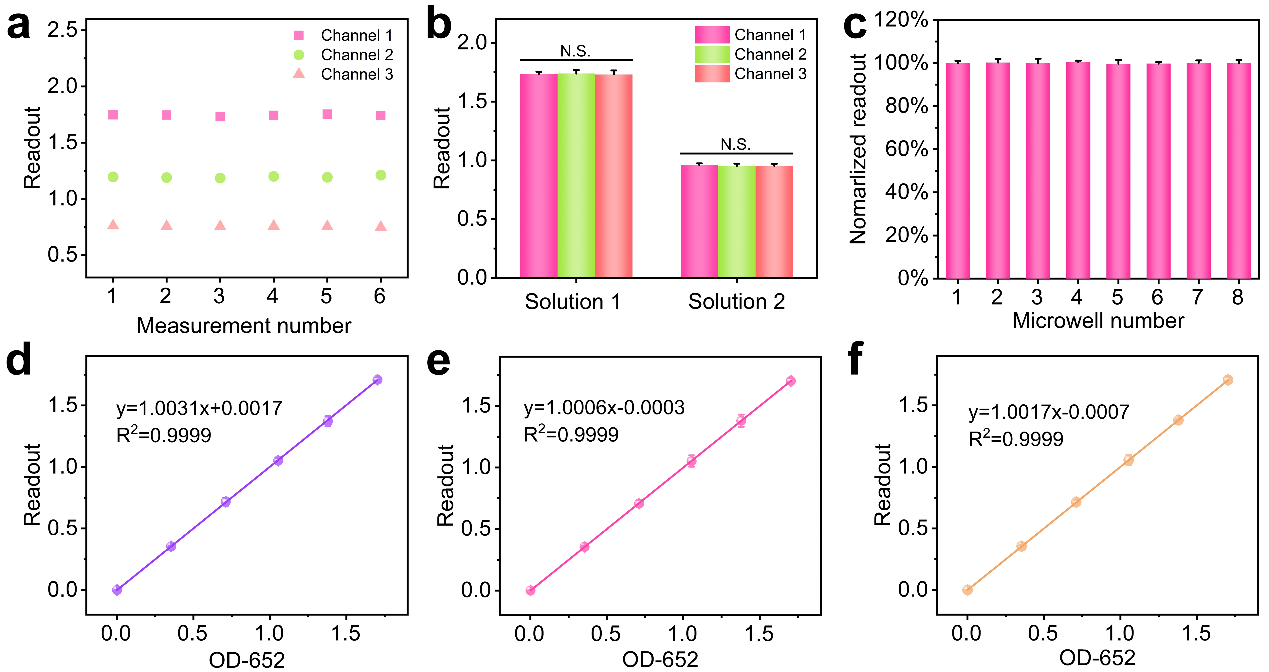
**

**Figure S27.** Evaluation of the portable electronic device. (a) Repetitive response of three channels for solutions at various concentrations (0.1 M HAc-NaAc buffer containing 10 mM H_2_O_2_, 1.0 mM TMB, and 5 μg mL^-1^ HEAs for Channel 1, 5 μg mL^-1^ HEAs for Channel 2, 2 μg mL^-1^ HEAs for Channel 3). (b) Differences in the measurement results of the three channels for solutions at the same concentration (0.1 M HAc-NaAc buffer containing 10 mM H_2_O_2_, 1.0 mM TMB, and 5 μg mL^-1^ HEAs for Solution 1, 2.5 μg mL^-1^ HEAs for Solution 2). The data are presented as mean ± SD (n = 3 independent samples). (c) Readouts from eight microwells of an ELISA strip containing the same solution. The data are presented as mean ± SD (n = 3 independent samples). Linear relationship between the OD-652 obtained by the microplate reader and the readout obtained by the portable device of (d) Channel 1, (e) Channel 2, (f) Channel 3. The data are presented as mean ± SD (n = 3 independent samples).

**
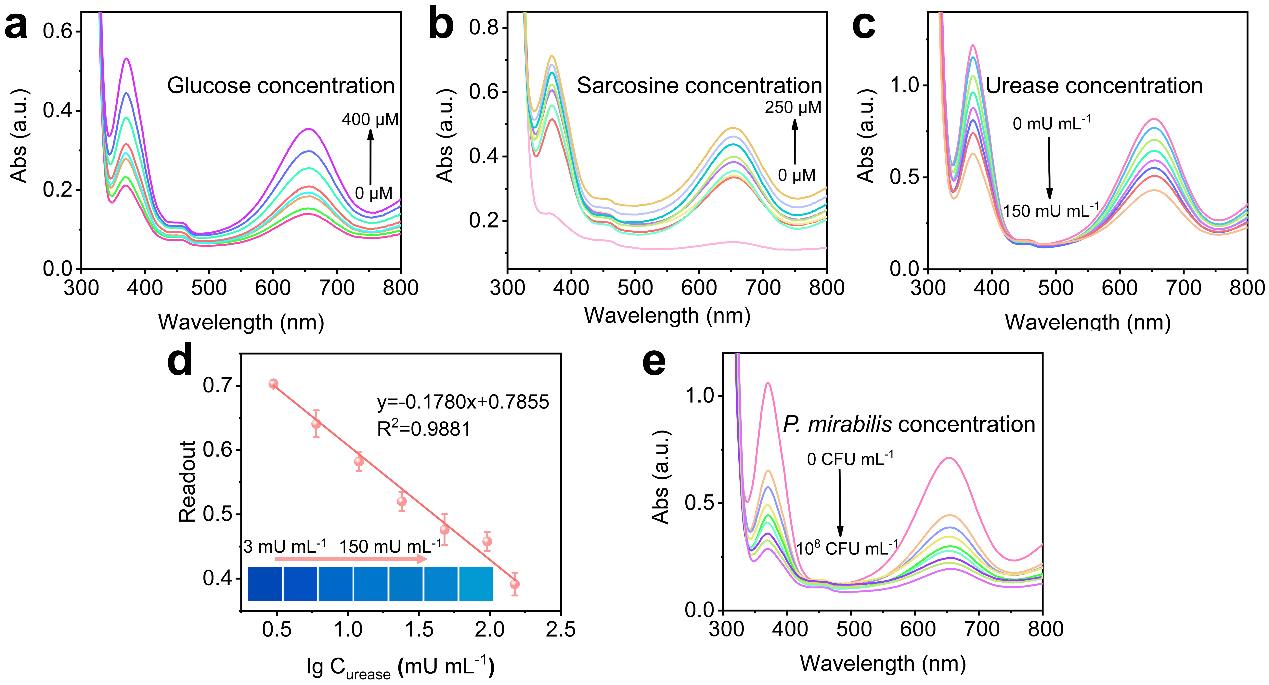
**

**Figure S28.** Detection of urinary biomarkers. UV-vis absorption spectra of (a) TMB/HEA NWs/GOX with glucose (0-400 μM), (b) TMB/HEA NWs/SOX with sarcosine (0-250 μM), (c) H_2_O_2_/TMB/HEA NWs/urea with urease (0-150 mU mL^-1^), and (e) H_2_O_2_/TMB/HEA NWs/urea with *P. mirabilis* (0-10^8^ CFU mL^-1^), respectively. (d) Calibration curves of the device readouts *v.s.* the lg concentration of urease in the H_2_O_2_/TMB/HEA NWs/urea system. Incubation condition: 10 min at 37 ºC, then 10 min at room temperature. The data are presented as mean ± SD (n = 3 independent samples).


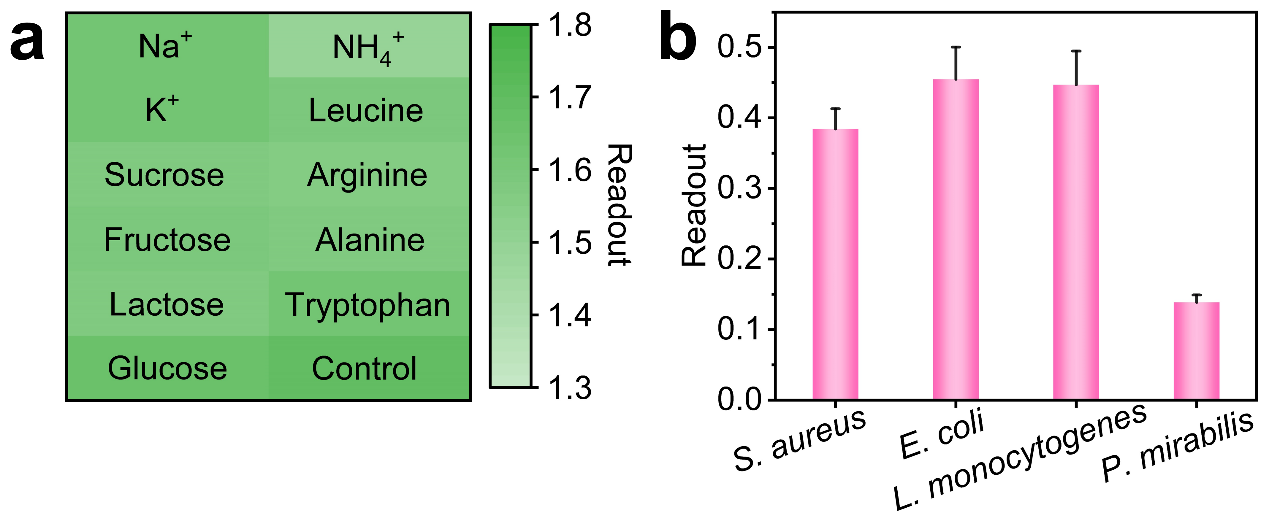


**Figure S29.** Selectivity analysis. (a) Selectivity assay of the HEA NWs/TMB chromogenic system. (b) The selectivity of the sensing platform for *P. mirabilis* detection.

**
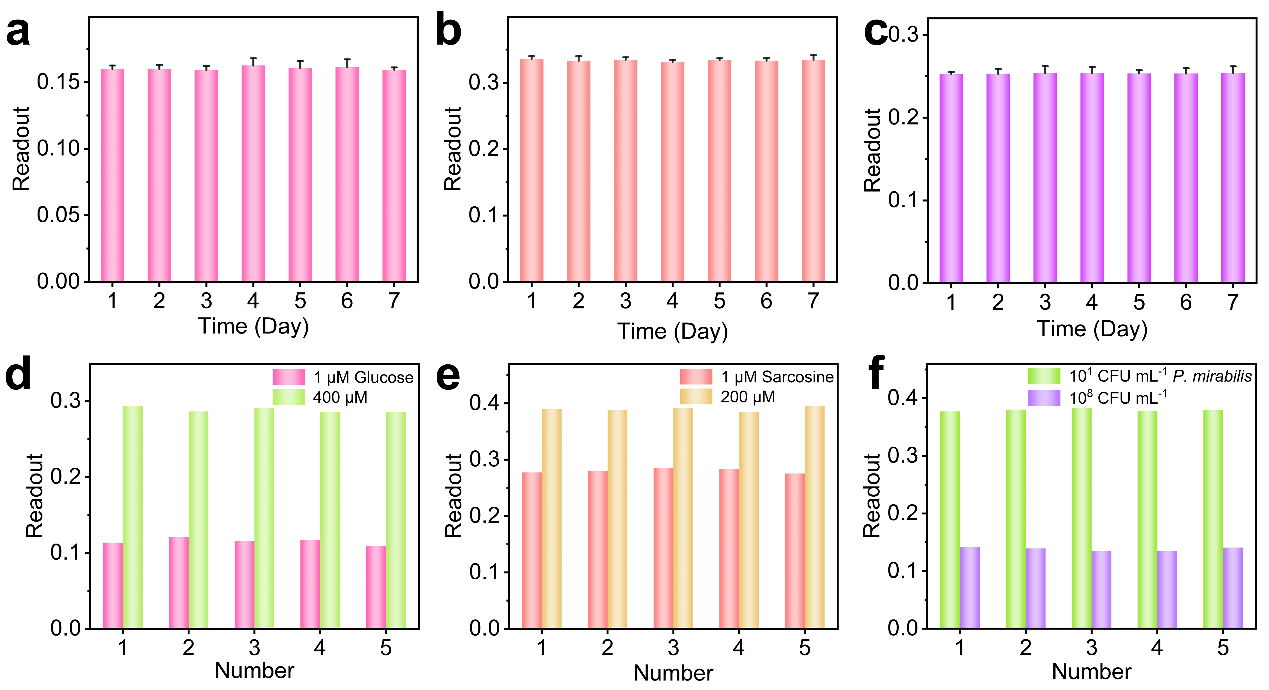
**

**Figure S30.** Assessment of stability and repeatability of detection. Detection stability of (a) glucose (100 μM), (b) sarcosine (100 μM), and (c) *P. mirabilis* (10^4^ CFU mL^-1^). The data are presented as mean ± SD (n = 3 independent samples). Detection repeatability of (d) glucose, (e) sarcosine, and (f) *P. mirabilis*.

**
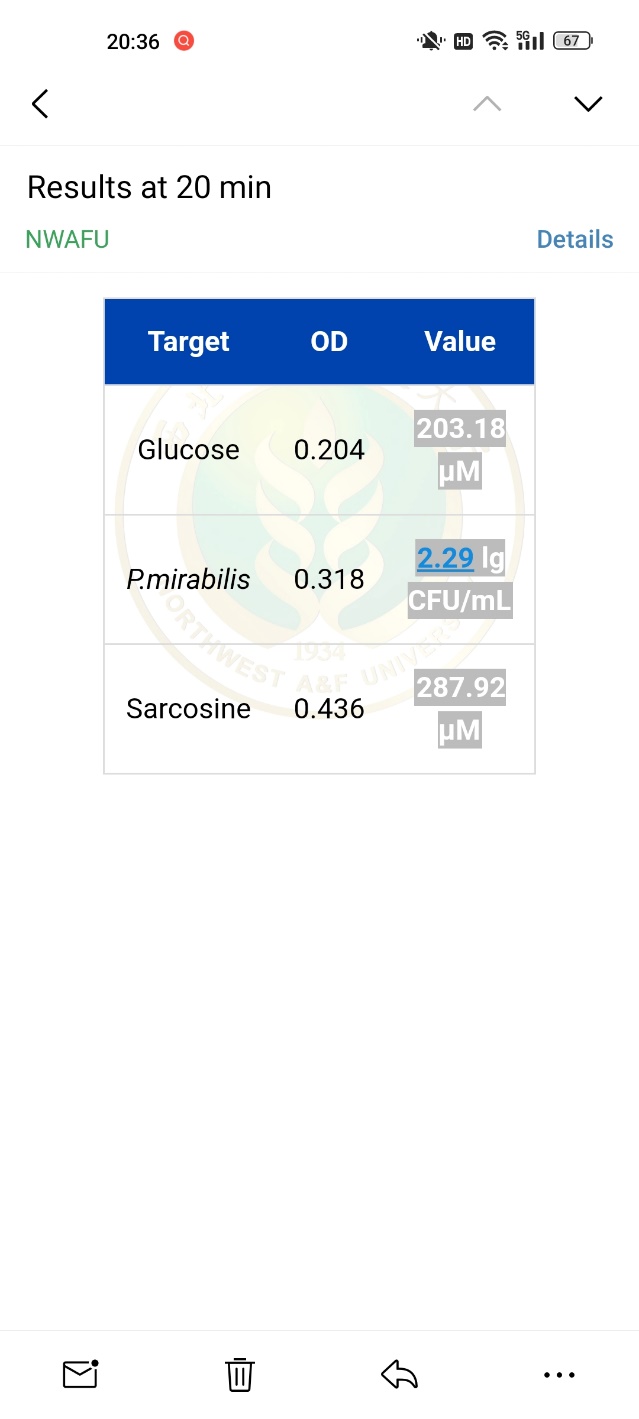
**

**Figure S31.** Detailed content of the E-mail. The calibration curve was built into the instrument and the email was designed to report the absorbance and the concentration of the target. The figure was a screenshot of the email in a real sample assay with spiked concentrations of 200 μM, 10^2^ CFU mL^-1^, and 250 μM for glucose, *P, mirabilis*, and sarcosine, respectively.
